# Low expression of the PPARγ-regulated gene thioredoxin-interacting protein accompanies human melanoma progression and promotes experimental lung metastases

**DOI:** 10.1038/s41598-021-86329-5

**Published:** 2021-04-12

**Authors:** Patrick Meylan, Christine Pich, Carine Winkler, Stefanie Ginster, Lionel Mury, Marie Sgandurra, René Dreos, Dennie Tompers Frederick, Marc Hammond, Genevieve Marie Boland, Liliane Michalik

**Affiliations:** 1 Center for Integrative Genomics, Faculty of Biology and Medicine, University of Lausanne, 1015 Lausanne, Switzerland; 2 Division of Surgical Oncology, Massachusetts General Hospital, Harvard Medical School, Boston, MA 02114 USA; 3 Present Address: Department of Dermatology and Venereology, University Hospital of Lausanne, Centre Hospitalier Universitaire Vaudois, Lausanne, Switzerland; 4 Present Address: R&D Philip Morris Products S.A, Neuchâtel, Switzerland

**Keywords:** Metastasis, Melanoma, Cell adhesion, Transcriptomics, Integrins, Nuclear receptors, Thioredoxins, Mouse, Computational biology and bioinformatics

## Abstract

The thioredoxin system plays key roles in regulating cancer cell malignancy. Here we identify the Thioredoxin-interacting protein (*TXNIP*) as a gene, which expression is regulated by PPARγ in melanoma cells. We show that high *TXNIP* expression levels associate with benign melanocytic lesions, with tumor regression in patients on MAP kinase targeted therapy, with decreased proliferation in patients’ melanoma biopsies, and with cell cycle arrest in human melanoma cell lines. In contrast, reduced *TXNIP* expression associates with advanced melanoma and with disease progression in patients. TXNIP depletion in human melanoma cells altered the expression of integrin beta-3 and the localization of the integrin alpha-v/beta-3 dimer at their surface. Moreover, TXNIP depletion affected human melanoma cell motility and improved their capacity to colonize mouse lungs in an in vivo assay. This study establishes *TXNIP* as a PPARγ-regulated gene in melanoma cells, thereby suggesting a link between these two proteins both involved in the regulation of cancer and of energy metabolism. It also reveals that the decrease in *TXNIP* expression, which is observed in advanced patient tumors, likely favors lung metastatic seeding of malignant cells.

## Introduction

Dissemination of malignant cells to distant organs is a lethal determinant in many cancers, including melanoma. Although much less frequent than skin basal-cell and squamous-cell carcinomas, skin melanoma remains the most aggressive form of skin cancers, responsible for 80% of skin cancer-related deaths, with incidence and associated mortality rates constantly increasing^1^. Although the landscape of metastatic melanoma treatment has dramatically changed over the past few years with the development of immuno- and molecularly targeted therapies, metastatic melanoma remains a tumor of dismal prognosis^2,3^. Therefore, it remains of critical importance to unearth the molecular mechanisms underlying melanoma progression and dissemination, not only to better understand the behavior of this tumor, but also to identify novel prognostic markers or targets with preventive or therapeutic potential.

The PPARγ nuclear hormone receptor is best known as a key regulator of energy metabolism and as the best-characterized molecular target of the insulin sensitizer drugs thiazolidinediones^4^. In addition, several studies have described beneficial roles of PPARγ agonists in melanoma cells through inhibition of proliferation, or activation of apoptosis and/or differentiation^5,6^. Pro-tumorigenic paracrine action of the PPARγ agonist rosiglitazone (RGZ) in melanoma has been reported, however^7,8^, showing that PPARγ activation results in complex and context-dependent outcomes, the molecular bases of which remain poorly characterized.

Thioredoxin-interacting protein (TXNIP, also named thioredoxin-binding protein 2 [TBP2] or vitamin D3 upregulated protein 1 [VDUP1]) is a ubiquitously expressed protein initially described as an endogenous inhibitor of thioredoxin expression and activity, which thereby counteracts thioredoxin protective role against oxidative stress^9^. Subsequently, TXNIP appeared to be a multifunctional protein, which has attracted attention as a regulator of lipid and glucose metabolism^10–12^. Several lines of evidence also suggest that the loss of TXNIP is involved in the progression of bladder and hepatocellular carcinoma^13^, while higher TXNIP levels are associated with a better prognosis in breast and gastric cancers, as well as in diffuse large B cell lymphoma^14–16^. While the last few years have provided considerable knowledge of TXNIP roles in the regulation of energy metabolism and associated pathologies, less is known regarding TXNIP contribution to the development of cancers.

The present study aims to elucidate TXNIP functions in human melanoma. Using bioinformatics analyses of public data, cell culture and in vivo models, we identify *TXNIP* as a PPARγ-regulated gene, the downregulation of which during melanoma progression likely contributes to malignant cell seeding to distant organs.

## Results

### The expression of *TXNIP* is regulated by PPARγ

**Figure 1:**
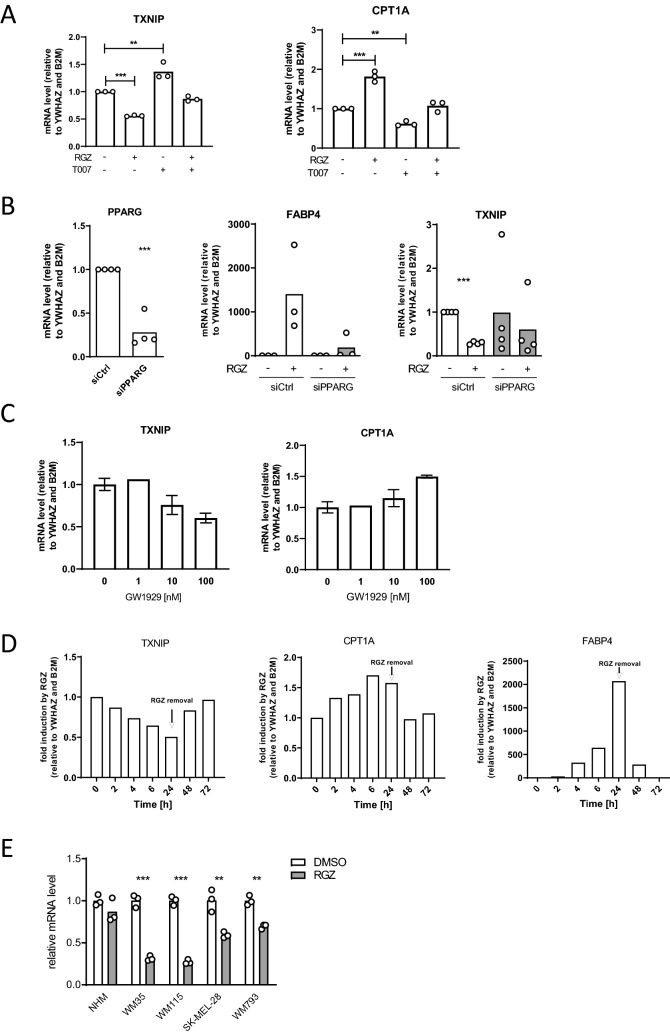
*TXNIP* is a PPARγ-regulated gene. **(A)** RT-qPCR analysis of *TXNIP* and *CPT1A* mRNA expression in A375 cells after a 24 h treatment with 5 μM of the PPARγ agonist RGZ, with 2 μM of the PPARγ antagonist T0070907 or both. House-keeping genes: *YWHAZ* and *B2M*. Each open circle represents one experiment with biological triplicates (n = 3). Columns are means of three independent experiments (N = 3). Statistics: one-way ANOVA with Dunnett’s multiple comparison test (**B**) RT-qPCR analysis of *PPARG*, *FABP4,* and *TXNIP* mRNA expression in A375 cells transfected with control (siCtl) or siPPARG siRNA, treated or not with 5 μM of the PPARγ agonist RGZ for 24 h, as indicated. House-keeping genes: *YWHAZ* and *B2M*. Each open circle represents one experiment with biological triplicates (n = 3). Columns are means of three to four independent experiments (N = 3–4). Statistics: unpaired Student’s t-test **(C)** RT-qPCR analysis of *TXNIP* and *CPT1A* mRNA expression in A375 cells after a 24 h treatment with increasing concentrations of the non-TZD PPARγ agonist GW1929. House-keeping genes: *YWHAZ* and *B2M*. Columns are means of biological duplicates (n = 2) of one experiment (N = 1) ± SD. **(D)** RT-qPCR analysis of *TXNIP, CPT1A, FABP4* mRNA expression in A375 cells after 2, 4, 6 and 24 h of 5 μM RGZ treatment, then 24 h, 48 h and 72 h after RGZ removal. House-keeping genes: *YWHAZ* and *B2M*. One time course experiment was performed (N = 1). Columns represent the means of technical replicates. **(E)** RT-qPCR analysis of *TXNIP* mRNA expression in normal human melanocytes (NHM) and in human melanoma cell lines WM35 (radial-growth phase), WM115 (vertical-growth phase), SK-MEL-28 (metastatic), and WM793 (metastatic) after a 24 h treatment with 5 μM RGZ. House-keeping genes: NHM: *TBP* and *B2M*; WM35: *YWHAZ* and *HPRT*; WM115: *TBP* and *HPRT*; SK-MEL-28: *YWHAZ* and *TFRC*; WM793: *YWHAZ*. Each open circle represents one experiment with biological triplicates (n = 3). Columns are means of three independent experiments (N = 3). Statistics: one-sample Student’s t-test per cell line. All panels: *, *P* < 0.05; ***, *P* < 0.001.

We first identified *TXNIP* as a PPARγ-regulated gene in a RNA-seq transcriptomic analysis of A375 human metastatic melanoma cell line described in^7^ (Primary data accession number: GSE115221). Briefly, A375 cells, which express high levels of PPARγ and exhibit the typical BRAFV600E mutations found in approximately 50% of patient tumors^2,17^, were treated with the PPARγ agonist Rosiglitazone (RGZ), the PPARγ antagonist T0070907 or a combination of both, in order to discriminate between true PPARγ-regulated genes and off-target effects of RGZ. Only the genes significantly deregulated by RGZ but not by RGZ in combination with T0070907 were considered as bona fide PPARγ-regulated genes and selected for further analysis. With this approach, we found that *TXNIP* expression was downregulated by PPARγ activation in A375 metastatic melanoma cells (absolute fold change −1.36; False discovery rate 0.031;^7^). We then validated PPARγ-dependent regulation of *TXNIP* with several independent approaches, using *CPT1A* or *FABP4* canonical targets as readouts for PPARγ activity. As expected for *bona fide* PPARγ-regulated genes, *TXNIP* downregulation and *CPT1A* upregulation by RGZ were both prevented by cotreatment with the selective PPARγ antagonist T0070907 (Fig. 1A), and the regulation of *TXNIP* and *FABP4* was prevented by siRNA-mediated down-regulation of PPARγ (Fig. 1B). As a further evidence, we found that the non-thiazolidinedione PPARγ agonist GW1929 also downregulated *TXNIP* and upregulated *CPT1A* in a dose-dependent manner, ruling out RGZ-specific regulation of *TXNIP* expression (Fig. 1C). A kinetic study showed that TXNIP mRNA expression was modulated as early as 2 h after RGZ addition and that it quickly returned to basal values upon ligand clearance (Fig. 1D, left panel), a kinetic also observed for *CPT1A* and *FABP4* (Fig. 1D, middle and right panels), two canonical direct PPARγ target genes. Finally, downregulation of *TXNIP* expression levels by RGZ was not specific to the A375 metastatic cells, as it was also observed in the human melanoma cell lines WM35 (radial-growth phase), WM115 (vertical-growth phase), SK-MEL-28 (metastatic) and WM793 (metastatic) human melanoma cells (Fig. 1E). In contrast, RGZ treatment had little impact on *TXNIP* expression in healthy primary human melanocytes (NHM, Fig. 1E).

### Low *TXNIP* expression associates with advanced melanocytic lesions, tumor progression, and higher proliferation in patients’ melanoma biopsies

**Figure 2:**
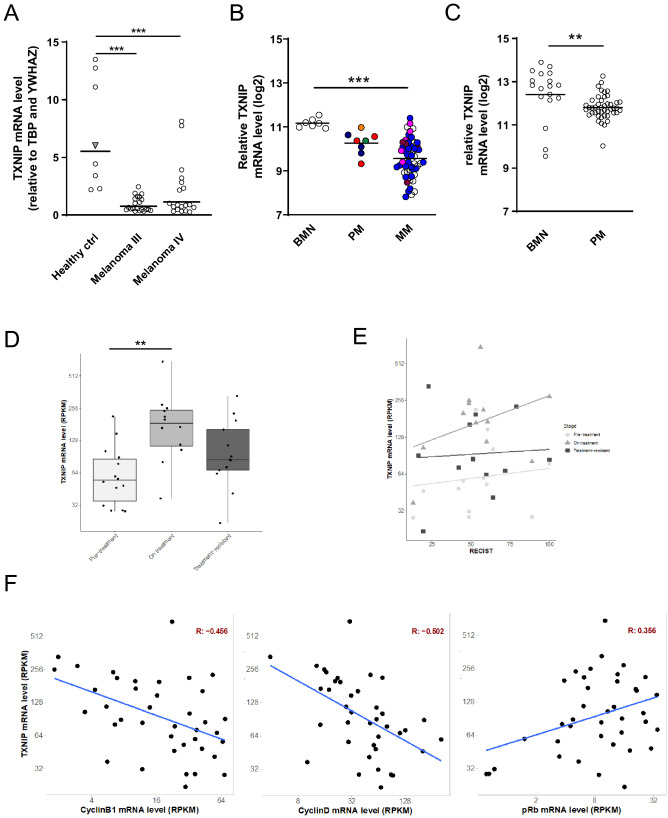
Low *TXNIP* expression levels associate with advanced melanocytic lesions, with tumor progression, and with higher proliferation in patients’ melanoma biopsies.** (A)** RT-qPCR analysis of *TXNIP* mRNA expression in healthy controls and human metastatic melanomas stage III and stage IV. Open circles represent individual donors; grey triangle represents a pool of primary melanocytes isolated from several donors; lines represent means. House-keeping genes: *TBP* and *YWHAZ*. Statistics: one-way ANOVA with Dunnett’s multiple comparison test. **(B)** Expression of *TXNIP* (log2 scale) in a microarray dataset (GSE46517) comparing 7 benign melanocytic nevi (BMN), 8 primary melanomas (PM) and 57 metastatic melanomas (MM). Circles represent individual samples; lines represent means. Colors represent tumor staging according to the AJCC staging system (8th edition, 2014): orange : stage 1A; green : stage 1B; dark blue : stage 2A; red: stage 2B; pink: stage 3B; burgundy: stage 3C; light blue: stage 4. Statistics: one-way ANOVA with Dunnett’s multiple comparison test. **(C)** Expression of *TXNIP* (log2 scale) in a microarray dataset (GSE 3189) comparing 18 BMN and 45 PM. Open circles represent individual samples; lines represent means. Statistics: unpaired Student’s t-test. **(D)** *TXNIP* expression values in Reads Per Kilobase of transcript per Million mapped reads (RPKM) stratified by tumor stages. Differences were analyzed using analysis of variance followed by Tukey test (*p*-value Pre-treatment versus On-treatment: 0.0014). **(E)** *TXNIP* expression values (RPKM) plotted against RECIST (Response Evaluation Criteria in Solid Tumors) scores stratified by tumor stage. Light grey circle: Pre-treatment; medium grey triangle: On-treatment; dark grey square: Treatment-resistant. Lines represent the regression analyses evaluated using linear models. A statistically significant coefficient was found for On-treatment samples (*P* = 0.0089). **(F)** Correlation analysis between *TXNIP* expression values (RPKM) and Cyclin B1 (*CCNB1*), Cyclin D (*CCND1*) and pRb (*RB1*). Correlation coefficient (R) is plotted in the top right corner, whereas regression lines calculated with liner models are plotted in blue (coefficients' *p*-values: *CCNB1* = 0.0035; *CCND1* = 0.00112; *RB1* = 0.0262). All panels: **, *P*  < 0.01; ***, *P* < 0.001.

Several studies have reported roles of PPARγ and its agonists in melanoma cells using in vitro and in vivo approaches^5^ but have in most cases not addressed the mechanisms involved. We thus sought to assess what contribution could TXNIP make to the roles played by PPARγ and its agonists in melanoma cells. As a first approach to a better understanding of TXNIP functions in melanoma, we quantified its expression in metastatic melanoma of commercially available melanoma cDNA arrays, as well as in two publicly available NCBI GEO microarray datasets (GSE46517 and GSE3189, chosen for high sample numbers and unequivocal experimental design) of benign melanocytic nevi (BMN), primary (PM) or metastatic (MM) melanomas. We found that *TXNIP* RNA levels were six times lower in melanoma as in healthy control samples (Fig. 2A), and also significantly downregulated in malignant (PM or MM) versus benign (BMN) lesions in the two microarray datasets analyzed (Fig. 2B,C; Supp. Figure 1A). We next addressed whether variations in *TXNIP* expression levels were associated with disease stabilization or regression upon MAP kinase targeted therapy. We used RNA-seq data from a unique set of patient metastases' biopsies that have been acquired longitudinally from patients with metastatic melanoma before (Pre-treatment) and during (On-treatment) MAP kinase targeted therapy, as well as treatment-resistant tumor biopsies (Treatment-resistant) (Table 1;^18^). Analysis of these data combined to RT-qPCR quantifications showed that On-treatment specimens expressed significantly higher levels of *TXNIP* compared to Pre-treatment specimens (Fig. 2D; Supp. Figure 1B). Interestingly, higher *TXNIP* expression levels in On-treatment specimens positively correlated with higher degree of tumor regression (RECIST, Response Evaluation Criteria in Solid Tumors; linear model, *P* = 0.00898; Fig. 2E). In the same patient specimen, we found an anti-correlation of *TXNIP* expression with the proliferation markers Cyclin B1 (*CCNB1)* and Cyclin D (*CCND1*) (Pearson correlation coefficient −0.456 and −0.502, respectively), and a correlation of *TXNIP* expression with the cell-cycle inhibitor pRb (*RB1 ;* retinoblastoma protein; Pearson correlation coefficient 0.356) (Fig. 2F). Along the same line, increased expression of *TXNIP* in On-treatment specimens was associated with decreased expression of the proliferation marker *PCNA* and lower proportion of Ki67-positive melanoma cells in 10 out of the 12 patient samples analyzed (Supp Fig. 1B–D).

**Table 1:** Patient clinical informations.

Patient ID#	Treatment	Pre-treatment biopsy (day)	On-treatment biopsy (day)	Progression biopsy (day)	Response (RECIST)
2	Vemurafenib^a^	−33	9	NA	PR (‐60.5%)
6	Dabrafenib^a^ + Trametinib^b^	−42	28	NA	PR (‐59.9%)
8	Dabrafenib^a^ + Trametinib^b^	−2	5	NA	PR (‐30%)
10	Dabrafenib^a^ + Trametinib^b^	0	12	NA	SD (−13%)
11	Dabrafenib^a^ + Trametinib^b^	−3	6	NA	PR (−80%)
12	Dabrafenib^a^ + Trametinib^b^	0	8	NA	PR (−88.9%)
16	Dabrafenib^a^ + Trametinib^b^	−9	7	335, off drug	SD (−19.5%)
24	Vemurafenib^a^	0	7	266, on drug	PR (−53%)
34	LGX818^a^ + MEK162^b^	−4	16	487, off drug	PR (−75.8%)
35	LGX818^a^ + MEK162^b^	−1	21	707, off drug	PR (−38.6%)
40	Vemurafenib^a^	−5	10	NA	PR (UN)
42	LGX818^a^ + MEK162^b^	0	27	496, off drug	PR (−76.1%)

*Biopsy day:* date of biopsy-therapy start date, *RECIST:* best response measured radiographically, *PR:* partial response, *SD:* stable disease, *UN:* unknown.

^a^BRAFi.

^b^MEKi.

### *TXNIP* expression and melanoma cell cycle arrest or apoptosis

**Figure 3:**
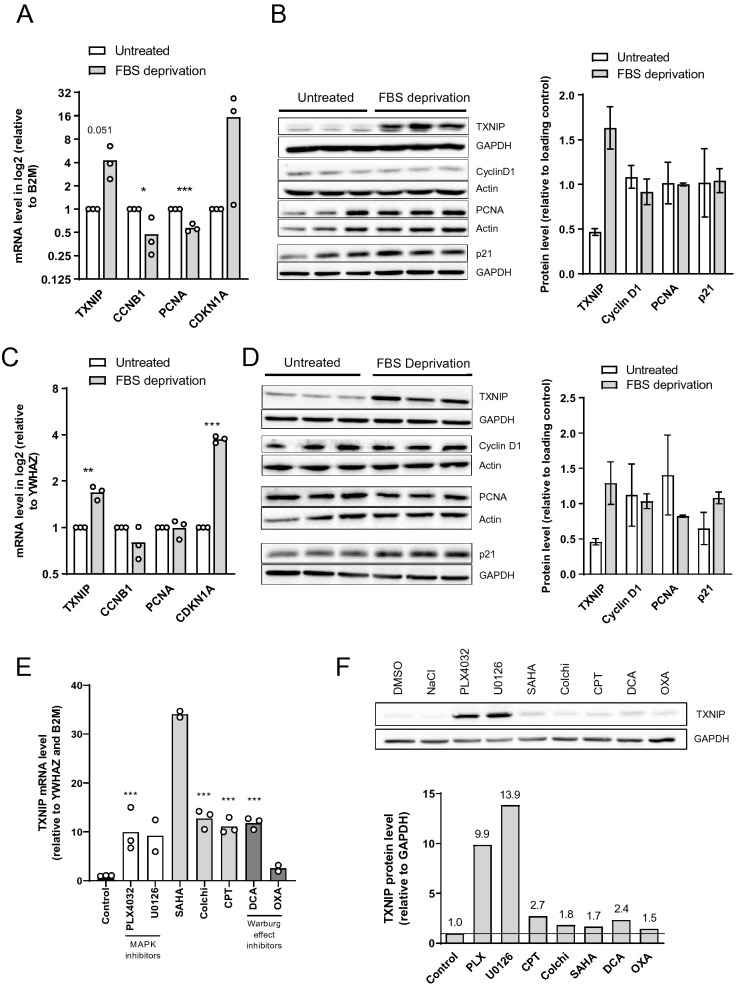
*TXNIP* upregulation is associated with decreased proliferation in human melanoma cells. **(A–C)** RT-qPCR analysis of *TXNIP*, *CCND1*, *PCNA* and *CDKN1A* mRNA levels (log2 scale) in human melanoma cells **(A)** A375 or **(C)** C8161 after 24 h of serum deprivation. Each open circle represents one experiment with biological triplicates (n = 3). Columns are means of three independent experiments (N = 3). House-keeping genes: *B2M* **(A)** and *YWHAZ* **(C)**. Statistics: unpaired Student’s t-test. **(B–D)** Left panels: western blot analysis of TXNIP, Cyclin D1, PCNA and p21 protein levels in human melanoma cells **(B)** A375 or **(D)** C8161 after 24 h of serum deprivation. Actin or GAPDH were used as loading controls and were run on the same gel as the protein of interest. Cropped western blots are shown. Full-length western blots and densitometry are shown in Supplementary Figs. 4 and 5. Right panels: quantification of western blot shown on left panels. Columns are means of biological triplicates (n = 3) ± SD. **(E)** RT-qPCR analysis of *TXNIP* mRNA levels in human A375 melanoma cells treated for 24 h with PLX4032 (BRAFV600E inhibitor; 100 nM), U0126 (MEK inhibitor; 5 mM), SAHA (a histone deacetylase inhibitor; 5 mM), colchicine (a microtubule polymerization inhibitor, 100 nM), CPT (camptothecin, a topoisomerase I inhibitor and anticancer compound; 1 mM), DCA and OXA (dichloroacetate; 50 mM and sodium oxamate; 10 mM, respectively, two inhibitors of the Warburg effect). The effect of each drug was normalized to its corresponding control; only one column is shown for controls, for the sake of simplicity. Each open circle represents one experiment with biological triplicates (n = 3). Three independent experiments were performed (N = 3) except for SAHA and OXA (N = 2). Columns are means of two to three independent experiments. House-keeping genes: *YWHAZ* and *B2M*. Statistics: one-way ANOVA with Dunnett’s multiple comparison test. **(F)** Top panel: western blot analysis of proteins extracted from human A375 melanoma cells treated for 48 h as in **(E).** Loading control (GAPDH) was run on the same gel. Cropped western blots are shown. Full-length western blot and densitometry are shown in Supplementary Fig. 6. Bottom panel: Quantification of the immunoblot shown in top panel (N = 1). Numbers represent fold changes to control treatments. All panels: *, *P* < 0.05; **, *P* < 0.01; ***, *P* < 0.001.

To substantiate the observation that higher *TXNIP* level was associated with decreased proliferation in metastatic melanoma biopsies, we analyzed the profile of *TXNIP* expression in proliferating and resting A375 cells, as well as in C8161 cells, a cutaneous metastatic melanoma cell line that also harbor an activating mutation of BRAF commonly found in patient tumors^17,19,20^. A375 and C8161 cells were serum-deprived to provoke cell cycle arrest. Like in patient melanocytic lesions, we observed an increase of TXNIP expression, concomitantly with a downregulation of the proliferation marker Cyclin D1 (A375, Fig. 3A,B) or an upregulation of the cell cycle inhibitor p21 (C8161, Fig. 3C,D). Moreover, we found that TXNIP was significantly upregulated at the mRNA and protein levels in A375 cells by a wide range of drugs known to have strong anti-proliferative activity via diverse mechanisms (Fig. 3E,F). Of note, the increase in TXNIP expression observed in A375 upon treatment with the MAP kinase pathway inhibitors (Fig. 3E,F) is in line with the increase in TXNIP expression that we uncovered in patients under MAP kinase targeted therapy (Fig. 2D; Table 1).

We next mimicked the decreased *TXNIP* expression that seemingly occurs in the course of melanoma progression. We derived fluorescent A375 cells constitutively expressing an shRNA against TXNIP (A375-shTXNIP) or a random shRNA (A375-shScr) using lentiviral transduction. We selected a polyclonal A375-shTXNIP cell population exhibiting a 30% decrease in *TXNIP* expression at the RNA level, in line with the magnitude of *TXNIP* downregulation that occurs in malignant (PM or MM) versus benign (BMN) lesions (Fig. 2B,C; Supp Fig. 2A, RNA and protein levels, left and right panels, respectively). This decrease in *TXNIP* RNA was accompanied by a 20% decrease in TXNIP protein (Supp Fig. 2A, right panel). We found that neither proliferation nor apoptosis of A375 cells was affected by decreased TXNIP expression levels (Fig. S2B,C, and D).

### A decrease in TXNIP expression alters the expression of genes involved in melanoma cell adhesion and in extracellular matrix remodeling

**Figure 4:**
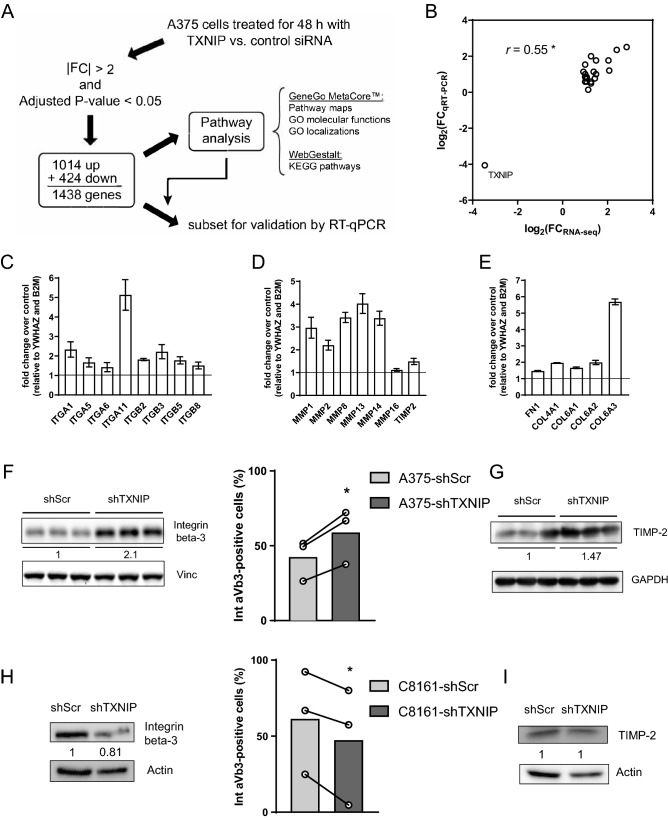
TXNIP knockdown affects the expression of genes involved in human melanoma cell adhesion and in extracellular matrix remodeling. **(A)** Overview of the pipeline used to analyze the RNA-seq of A375 cells under transient TXNIP knockdown. FC, fold change. **(B)** Spearman’s correlation plot comparing fold changes measured by RT-qPCR and by RNA-seq (analysis of three independent experiments, N = 3) for the panel of genes shown in **(C–E)** RT-qPCR analysis of TXNIP knockdown-induced changes in the mRNA expression of the genes selected for validation. Columns are means of two biological duplicates (n = 2) ± SD of one RT-qPCR quantification. House-keeping genes: *YWHAZ* and *B2M.* **(F)** Left panel: Western blot analysis of integrin beta-3 levels in total proteins extracted from A375-shScr and A375-shTXNIP; vinculin (Vinc) was used as a loading control and was run on the same gel; biological triplicates (n = 3) from one experiment are shown. Cropped western blots are shown; full-length western blots and densitometry are shown in Supp. Figure 7. Right panel: Flow cytometry analysis of integrin alpha-v/beta-3 (Int av/b3) surface expression in A375-shScr and A375-shTXNIP cells. Each open circle represents one independent experiment with biological duplicates (n = 2). Columns are means of three independent experiments (N = 3). Statistics: paired Student’s t-test. **(G)** Western blot analysis of tissue inhibitor of metalloproteinase-2 (TIMP-2) protein levels in total proteins extracted from A375-shScr and A375-shTXNIP cells; GAPDH was used as a loading control and was run on the same gel; triplicates (n = 3) from one experiment are shown. Cropped western blots are shown; full-length western blots and densitometry are shown in Supplementary Fig. 7. **(H,I)** Western blot analysis of Integrin beta-3 (H, left) and TIMP2 (I) in total proteins extracted from C8161-shScr and C8161-shTXNIP cells; Actin was used as loading control and was run on the same gel. Cropped western blots are shown; full-length western blots and densitometry are shown in Supplementary Fig. 7. **(H)** Right: Flow cytometry analysis of integrin alpha-v/beta-3 (Int av/b3) surface expression in C8161-shScr and C8161-shTXNIP cells. Each open circle represents one independent experiment with biological duplicates (n = 2). Columns are means of three independent experiments (N = 3). Statistics: paired Student’s t-test. All panels: *, *P* < 0.05; ***, *P* < 0.001.

To shed light onto the potential roles of TXNIP in human melanoma cells, we performed an RNA-seq on A375 cells transiently transfected with TXNIP versus control siRNA (Supp Fig. 2E). Transient knockdown was chosen here to identify the direct effects of the TXNIP knockdown. With cut-off values for adjusted *P*-value and absolute fold change set at 0.05 and 2 respectively, we obtained 1438 significantly deregulated (1014 up- and 424 downregulated) genes (Fig. 4A; Table 2 for the top ten downregulated and upregulated genes). The two protein classes most significantly affected by TXNIP knockdown were ligands (enrichment ratio = 1.9; *P* < 0.001) and receptors (1.7; *P* < 0.001) (Table S1). Gene ontology (GO) analyses were in line with this finding; the three most significant GO localizations were “Cell periphery”, “Plasma membrane” and “Plasma membrane part” (Table S2), and the three most significant GO molecular functions were “Protein binding”, “Binding” and “Receptor binding” (Table S3). Interestingly, the three most significantly deregulated pathway maps included “Cell adhesion extracellular matrix remodeling” and “Development and regulation of epithelial-to-mesenchymal transition” (Table S4). Moreover, several of the most deregulated KEGG pathways were related to cell adhesion (Table S5), overall suggesting a potential role of TXNIP in melanoma cell dissemination.

**Table 2:** Top 10 significantly downregulated (upper part) and upregulated (lower part) genes upon TXNIP knockdown in A375 cells, as measured by RNA-seq.

Ensembl Gene ID	Gene symbol	Gene name	Adjusted *P*-value	Fold change
**Downregulated**				
ENSG00000182795	C1orf116	Chromosome 1 open reading frame 116	0.0054	−13.2
ENSG00000117289	TXNIP	Thioredoxin interacting protein	0.0054	−11.0
ENSG00000143631	FLG	Filaggrin	0.0061	−10.2
ENSG00000033122	LRRC7	Leucine rich repeat containing 7	0.0070	−9.3
ENSG00000163347	CLDN1	Claudin 1	0.0054	−8.0
ENSG00000182636	NDN	Necdin	0.0057	−7.8
ENSG00000143473	KCNH1	Potassium channel, voltage gated eag related subfamily H, member 1	0.0058	−6.7
ENSG00000184838	PRR16	Proline rich 16	0.0061	−6.4
ENSG00000189068	VSTM1	V-set and transmembrane domain containing 1	0.0054	−6.2
ENSG00000148677	ANKRD1	Ankyrin repeat domain 1 (cardiac muscle)	0.0057	−5.9
**Upregulated**				
ENSG00000133067	LGR6	Leucine-rich repeat containing G protein-coupled receptor 6	0.0054	18.7
ENSG00000162511	LAPTM5	Lysosomal protein transmembrane 5	0.0054	16.2
ENSG00000231574	KCCAT211	Renal clear cell carcinoma-associated transcript 211	0.011	13.2
ENSG00000229162	RP11-84D1.1		0.0057	8.2
ENSG00000187634	SAMD11	Sterile alpha motif domain containing 11	0.0057	7.9
ENSG00000163359	COL6A3	Collagen, type VI, alpha 3	0.0054	7.2
ENSG00000104435	STMN2	Stathmin 2	0.0054	6.4
ENSG00000255346	NOX5	NADPH oxidase, EF-hand calcium binding domain 5	0.010	6.3
ENSG00000054356	PTPRN	Protein tyrosine phosphatase, receptor type, N	0.0054	6.3
ENSG00000224596	ZMIZ1-AS1	ZMIZ1 antisense RNA 1	0.0054	6.0

We next selected a subset of significantly deregulated genes with functional annotations related to cell adhesion and extracellular matrix (ECM) remodeling for further validation. This subset comprised three categories of genes: integrins, matrix metalloproteases (MMPs) and ECM components. For all the selected genes except *MMP16*, fold changes measured by RT-qPCR were tightly correlated (r = 0.94; *P* < 0.001) with those measured by RNA-seq (Fig. 4B–E). Among those actors of particular interest to melanoma metastasis, a strong increase in the expression of integrin beta-3 (*ITGB3*) and TIMP-2 was confirmed at the protein level in A375-shTXNIP versus A375-shScr human melanoma cells (Fig. 4F–G), while integrin alpha-11, collagen alpha3(VI), MMP-1 and MMP-14 proteins remained unaffected (Supp Fig. 3A–D). A FACS analysis revealed that the increased expression of integrin beta-3 was associated with a significant upregulation of the integrin alpha-v/beta-3 dimer at the surface of A375-shTXNIP compared to A375-shScr cells (Fig. 4F, right panel). Integrin beta-3 expression level and alpha-v/beta-3 localization at the cell membrane were also affected upon shRNA-mediated TXNIP silencing in C8161 cells; in those cells, however, TXNIP silencing (Supp Fig. 3E) resulted in a downregulation of integrin beta-3 expression and alpha-v/beta-3 localization at the cell membrane, while TIMP2 expression remained unaffected (Fig. 4H–I).

### TXNIP knockdown affects melanoma cell adhesion and impairs their migration

**Figure 5:**
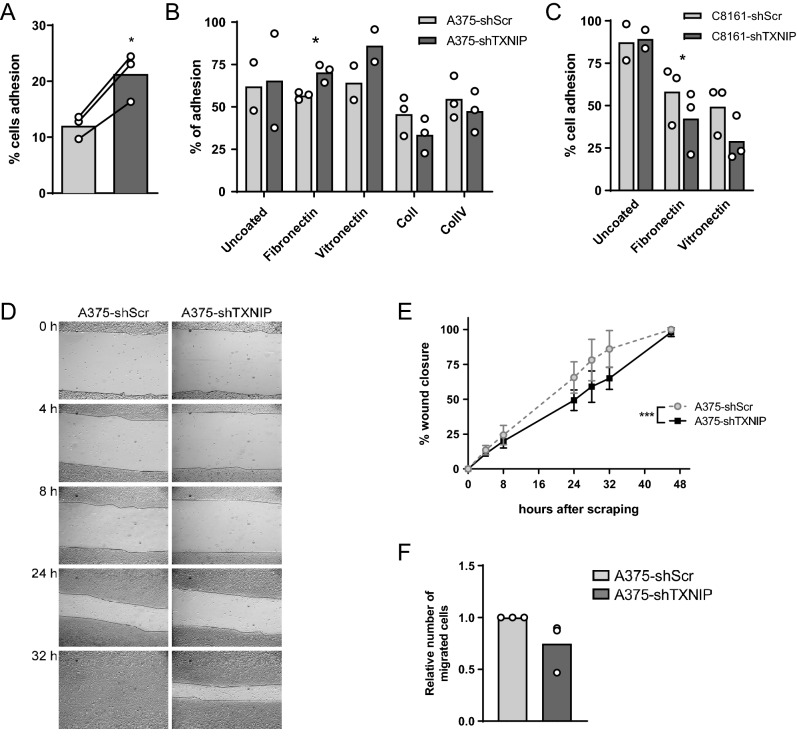
TXNIP silencing affects melanoma cell adhesion and migration. **(A)** Trypsin resistance assays of A375-shScr and A375-shTXNIP cells. Each open circle represents one independent experiment with biological triplicates (n = 3). Columns are means of three independent experiments (N = 3). Statistics: paired Student’s t-test. **(B)** Adhesion assays of A375-shScr and A375-shTXNIP cells on various ECM protein coatings, as indicated. Each open circle represents one independent experiment with biological triplicates (n = 3). Except for Uncoated and Vitronectin treatments (N = 2), three independent experiments were performed (N = 3). Columns are means of two to three independent experiments. Statistics: paired Student’s t-test. **(C)** Adhesion assays of C8161-shScr and C8161-shTXNIP cells on various ECM protein coatings, as indicated. Each open circle represents one independent experiment with biological triplicates (n = 3). Columns are means of two to three independent experiments (N = 3). Statistics: paired Student’s t-test. **(D)** A375-shScr and A375-shTXNIP cell monolayer scraping assays. The quantification of three independent scraping assays (N = 3) is shown in **(E)** Grey circles/dotted line and black squares/plain line represent the means of three independent experiments (N = 3); each independent experiment included biological triplicates (n = 3). Data are expressed as means ± SD. Statistics: two-way ANOVA **(F)** Number of A375-shScr and A375-shTXNIP cells migrating through a porous membrane in a transwell assay, 6 h after plating. Each open circle represents one independent experiment with biological triplicates (n = 3). Columns are means of three independent experiments (N = 3). All panels *, *P* < 0.05; **, *P* < 0.01.

We next examined the functional impact of TXNIP-dependent regulation of genes involved in melanoma cell adhesion and migration—two critical processes for metastasis formation. TXNIP-dependent regulation of human melanoma cell adhesion was assessed using trypsin resistance assays as well as conventional adhesion assays. In the former, A375-shTXNIP cells demonstrated significantly more resistance to trypsin-mediated cell–matrix dissociation (Fig. 5A). In agreement with an active role of TXNIP in controlling melanoma cell adhesion, conventional adhesion assays revealed enhanced adhesion of A375-shTXNIP cells on plates coated with fibronectin (Fig. 5B), while C8161-shTXNIP cells exhibited decreased adhesion on the same substrate (Fig. 5C). A375-shTXNIP cells also tended to display better adhesion than A375-shScr cells on vitronectin (Fig. 5B). Additionally, cell monolayer scraping (Fig. 5D,E) and transwell migration assays (Fig. 5F) revealed that A375-shTXNIP cells migrated significantly less efficiently than A375-shScr cells. As described above (Supp Fig. 2), proliferation and apoptosis rates were not affected by TXNIP knockdown, ruling out any bias in the assessment of migration speed.

### Decrease in TXNIP expression levels favor melanoma cell seeding to the lung

**Figure 6:**
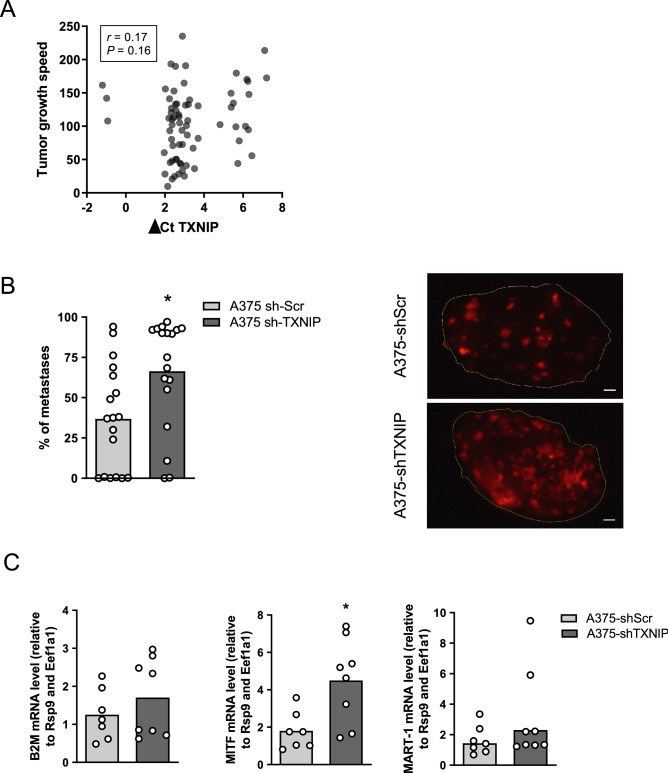
TXNIP silencing favors experimental human melanoma cell seeding into the lung. **(A)** Pearson correlation plot of subcutaneous tumor growth speed (mm^3^ per day) and *TXNIP* mRNA expression in the tumor at the time of tumor collection. Each circle represents one individual tumor. **(B) **Left panel: Whole mount lung quantification of tumors three weeks after intravenous injection of mCherry-labeled A375-shScr or A375-shTXNIP cells in NSG mice. Each circle represents one individual mouse. Columns are means of individual samples (N = 18 mice per group). Statistics: unpaired Student’s t-test. Right panel: Representative pictures of lung surfaces collected three weeks after intravenous injection of mCherry-labeled A375-shScr or A375-shTXNIP in NSG mice (N = 18 mice per group). Scale bar: 1 mm. **(C)** RT-qPCR analysis of mRNA relative expression of the human genes *B2M, MITF* and *MART-1* over the murine house-keeping genes *Rsp9* and *Eef1a1* in lungs collected 3 weeks after intravenous injection of mCherry-labeled A375-shScr or A375-shTXNIP cells in NSG mice. Each open circle represents one individual mouse (N = 7 A375-shScr mice; N = 8 A375-shTXNIP mice). Columns represent means of individual samples. Statistics: unpaired Student’s t-test. All panels *, *P* < 0.05.

We next assessed the impact of TXNIP downregulation in melanoma cells in vivo using only A375 metastatic cells as a model in order to limit animal use. The above-described A375-shTXNIP or A375-shScr human melanoma cells were implanted subcutaneously in immunocompromised mice. In line with our observations that a decrease in TXNIP expression did not affect melanoma cell proliferation nor apoptosis, we found no correlation between TXNIP expression levels in the melanoma xenografts and their growth rate (Fig. 6A). We next addressed TXNIP role in melanoma cell seeding to distant organs by quantifying the ability of fluorescently-labelled TXNIP-deficient A375 cells to establish lung tumors following injection into the tail vein of immunocompromised mice. Quantitative assessment of tumors of human-cell origin was performed by visual quantification of fluorescently labelled tumors in whole lungs (Fig. 6B). Quantification of fluorescence was complemented by an independent, RT-qPCR-based approached, in which we chose human *B2M, MITF* and *MART-1* as human cell markers quantified over two murine housekeeping genes to detect human cell presence in mouse lungs (Fig. 6C). Human *B2M* was chosen as a gene expressed in all human cells, while human *MITF* and *MART-1* were chosen as human melanocyte-lineage specific markers. Their expression was not significantly affected by TXNIP silencing in A375-shTXNIP cells, and amplification specificity of human *B2M, MITF* and *MART-1* was first verified by the absence of amplification of these cDNA in normal lung tissue of non-injected mice (data not shown). We found that the lung metastatic load was greater in mice injected with A375-shTXNIP compared to A375-shScr cells, as shown by greater number of fluorescently labelled tumors quantified in whole lungs and greater average expression of human *B2M and MITF* in the lung of these animals (Fig. 6B,C, respectively). Although not statistically significant, the expression of *MART-1* showed the same trend (Fig. 6C, right panel). TXNIP being known to play a role in melanoma cell intravasation via a redox-sensitive mechanism^21^, we addressed whether the cell seeding-promoting role of low TXNIP expression relied on a modulation of melanoma cell ROS content. ROS-sensitive fluorescence assays showed that knockdown of TXNIP did not affect H_2_O_2_-induced oxidative stress in A375 cells (Fig. S3G).

## Discussion

Our study reveals that a progressive decrease in *TXNIP* expression is associated with the progression of melanoma towards a metastatic phenotype, whereas high *TXNIP* expression level associates with tumor regression (RECIST) and with decreased proliferation in patients on MAP kinase targeted therapy. The contribution of TXNIP to the progression of melanoma is unknown, despite evidence of TXNIP-dependent regulation of proliferation, ROS levels and intravasation in mouse or human melanoma cells^21–23^. Furthermore, the majority of studies of TXNIP roles in cancers have investigated the consequences of its overexpression^13^, while interrogating the contribution of TXNIP downregulation to cancer progression has been neglected. Here we establish that TXNIP silencing in human melanoma cells—to an extent commensurate with the decrease observed in malignant versus benign lesions in patients—did not affect melanoma cell proliferation nor ROS levels but affected the expression of proteins of particular relevance to melanoma cell invasiveness, integrin alpha-v/beta-3 and TIMP-2. Experimental and endogenous upregulation of integrin beta-3 expression indeed correlates with the malignant potential of human melanoma cells, with the transition from dysplastic nevi to invasive melanomas and with preferential metastatic seeding to the lung ; conversely, normal melanocytes or early-stage melanomas do not express integrin beta-3^24,25^. More particularly, the activation of integrin alpha-v/beta-3 dimer is a characteristic of invasive melanoma cells^26–30^. Finally, TIMP-2 was shown to contribute to the activation of MMP-2 at the cell membrane in vitro and in vivo, and may thereby promote melanoma cell invasiveness^31,32^.

We further demonstrate that changes in the expression of genes involved in melanoma cell invasiveness has functional consequences: it affects cell adhesive and migratory properties and favors experimental metastatic seeding in mouse lungs in vivo. Consistent with our findings are the previous observations that TXNIP artificial overexpression in human melanoma cells reduces metastatic seeding in the lung of athymic mice^23^, and that miRNA-mediated TXNIP silencing in human breast cancer cells promotes metastasis formation^33^. Although TXNIP may exert anti-cancer functions through a variety of mechanisms, such as inhibition of proliferation, promotion of apoptosis, or reduction of ROS levels^9,10, 13,34^ our data indicate that in melanoma cells, decreased TXNIP expression may contribute to melanoma progression by promoting melanoma cell seeding to distant organs rather than fostering primary tumor growth.

On the basis of the beneficial impacts of its overexpression, TXNIP has been mentioned as potential therapeutic targets in cancers^13^. Our study suggests that in melanoma, reactivation of TXNIP expression may be of interest to reduce malignant cell invasive capacities. There are, however, important limitations to consider regarding the activation of TXNIP. First, we show that the downregulation of TXNIP had opposite effects on the expression of integrin alpha-v/beta-3 and TIMP-2 in two different human metastatic cell lines. This suggests context-dependent consequences in modulating TXNIP activity in melanoma, the characterization of which requires further investigation. Second, while activating TXNIP may be beneficial in cancer, data accumulated in the last few years strongly suggests that this would have a deleterious impact on lipid and glucose metabolism, with an increased risk of developing diabetes^11,12^. Thus, developing TXNIP-targeted therapy should be restricted to cancers exhibiting TXNIP downregulation and should aim at normalizing but not at increasing TXNIP activity.

Our study is the first to link TXNIP to another therapeutic target, the nuclear hormone receptor PPARγ. PPARγ is indeed the best-characterized target of the insulin-sensitizers of the thiazolidinedione family used in the treatment of type 2 diabetes, and its use as a therapeutic anti-cancer target is also a matter of debate. While PPARγ activation was mostly reported to exhibit anticancer effects in a variety of cancer cells including melanoma^35^, we have recently characterized a protumorigenic paracrine role of the PPARγ agonist RGZ in human melanoma^7,8^. Here we reveal that PPARγ activation reduces the expression of TXNIP in human melanoma cells. Whether the regulation of *TXNIP* by PPARγ is direct (that is, by direct binding of PPARγ in its promoter) or indirect goes beyond the objectives of this study and would require further investigation. Nevertheless, while this adds to the potentially protumorigenic effects of activating PPARγ in melanoma, it also raises the intriguing possibility that decreasing the expression of TXNIP, which is currently considered a promising approach for treating type 2 diabetes^11,12^, could be involved in PPARγ and RGZ-antidiabetic actions.

## Material and methods

### Surgical specimens

All experimental protocols involving surgical specimens were approved by ethics committees and research was performed in accordance with relevant regulations:

—Abdominal skin biopsies were obtained anonymously from the Department of Musculoskeletal Medicine Biobank, University Hospital of Lausanne, Switzerland. Informed consent for research was obtained prior to surgery and was regulated through the Department of Musculoskeletal Medicine Biobank (University Hospital of Lausanne, Switzerland, Profs. Raffoul and Applegate). The protocol was approved by the Canton de Vaud Ethics Committee on research involving humans (CER-VD).

—Serial melanoma metastases biopsies and clinical data were collected from patients with metastatic melanoma at the Division of Surgical Oncology, Massachusetts General Hospital, Boston, USA. All human subjects and associated materials were consented under the translational protocol approved by the Dana Farber/Harvard Cancer Center Institutional Review Board (IRB ; DFCI 11-181 ; PI: Boland).

Melanoma cDNA array (MERT501) containing ten identical sets of 43 tissues covering four disease stages (1 × Stage IIIA, 6 × IIIB, 4 × IIIC, 10 × III 19 × IV) and normal tissues was purchased from OriGene.

### Cell culture and treatments

Human melanoma cell lines A375 (CLS Cat# 300110/p852_A-375, RRID:CVCL_0132) and C8161 (RRID:CVCL_6813; kindly provided by Prof. D. Constam, EPFL, Lausanne, Switzerland) were grown in DMEM supplemented with 10% FBS. Pooled human healthy primary melanocytes (ATCC) and primary melanocytes from individual donors (kindly provided by Prof. D. Fisher (Massachusetts General Hospital, Cutaneous Biology Research Center, Boston, USA) were grown in Melanocyte Growth Medium M2 supplemented with SupplementMix Melanocyte Growth Medium (PromoCell). All cultures were maintained at 37 °C in a 5% CO_2_ humidified atmosphere, with medium renewal every 2 or 3 days.

A375 cells were treated during 24 h with PLX4032 (100 nM; Axon Medchem), U0126 (5 μM; New England Biolabs), suberoylanilide hydroxamic acid (5 μM), colchicine (100 nM), camptothecin (1 μM), dichloroacetate (50 mM) and sodium oxamate (10 mM) all from Sigma-Aldrich. The effect of each drug was normalized to its corresponding control: the vehicle for PLX4032, U0126, suberoylanilide hydroxamic acid and camptothecin was DMSO, used alone in control cells at a maximal concentration of 0.1% (vol/vol); an equimolar sodium chloride solution was used as a control for colchicine, dichloroacetic acid and sodium oxamate treatments.

### TXNIP knockdown

siTXNIP transfection: 20–50% confluent A375 were transfected with predesigned 27-mer siRNA duplexes (Origene) using Lipofectamine RNAiMAX (Life Technologies) as per manufacturer’s instructions, and incubated for 48 h at 37 °C. Scrambled siRNA (siControl) were used as controls.

shTXNIP transduction: A375 and C8161 were transduced with premade lentiviruses (GeneCopoeia) expressing TXNIP shRNA or scrambled control shRNA along with the mCherry fluorescent protein, using 5 μM polybrene (Sigma-Aldrich). 4 days later, medium supplemented with 1.25 μg/ml puromycin (InvivoGen) was added for at least 3 weeks for selection of transduced cells . No clonal selection was performed. Polyclonal batch cultures of A375-shTXNIP, A375-shScr, C8161-shTXNIP, C8161-shScr cells were checked for TXNIP expression levels by RT-qPCR and used for further experiments.

### Cell functional assays

Cell cycle arrest was provoked by serum deprivation (DMEM 0% FBS) for 24 h.

Cell monolayer scraping assays: cell monolayers were wounded by scratching with a plastic cell scraper trimmed (1 mm width). Nine random locations (3 per well of a 6-well plate, 3 wells per condition) were chosen for each condition where pictures were taken at the time points indicated in the figures. Wound area was measured on each picture using ImageJ and the percent wound closure calculated.

Cell adhesion assays: for trypsin resistance assays, A375-shTXNIP and A375-shScr cells grown on uncoated 24-well plates were washed with PBS and trypsinized for half the time normally required to detach all the cells. After washes, 50 μl RIPA was added to lyse the cells that were still attached. Wells where no trypsin was added were used to calculate the fraction of adhesive cells. For adhesion assays, 60 mm dishes were coated with collagen I (48 μg/ml), collagen IV (33 μg/ml), fibronectin (10 μg/ml) all from Sigma-Aldrich or vitronectin (3 μ/ml ; Thermo Fischer Scientific). A375-shTXNIP, A375-shScr, C8161-shTXNIP, and C8161-shScr cells were plated in each dish and incubated at 37 °C for 45 min. After two washes with PBS, adhesive cells were trypsinized and counted, and the fraction of adhesive cells calculated.

Transwell migration assays: A375-shScr and A375-shTXNIP cells were plated on cell culture microporous membranes (8 μm pore size, 1.10^5^ pores/cm^2^; Falcon/Brunschwig). They were then incubated at 37 °C / 5% CO_2_ in DMEM 0%FBS for 6 h with DMEM 10% FBS in the bottom well as chemoattractant. Medium was then removed from the inserts and from the companion plate wells. Cells that had not migrated were removed from the top of the membranes. Cells at the bottom of the membrane and in the wells were fixed (ice-cold methanol 100%; 10 min, RT) then stained with Crystal violet 0,5% for 10 min at RT. Cell quantification was performed using ImageJ.

### Flow cytometry analysis of integrins

A375-shScr, A375-shTXNIP, C8161-shTXNIP, and C8161-shScr cells were collected with Hank's based cell dissociation buffer (Life Technologies) and washed in PBS.

For integrin analysis, cells were stained with Alexa-Fluor 647 conjugated anti-integrin alpha-v/beta-3 mAb and isotope control (1:25 dilution, BioLegend Cat# 400130, RRID:AB_2800436) at 4 °C for 35 min, protected from light. Stained cells were analyzed on a Accuri C6 flow cytometer (BD Accuri C6 Plus, RRID:SCR_014422) and the results were analyzed with FlowJo software (RRID:SCR_008520). Percent of integrin alpha-v/beta-3 positive A375-shTXNIP, A375-shScr, C8161-shTXNIP, and C8161-shScr cells were quantified.

### RNA isolation and quantitative real-time PCR (RT-qPCR)

Total RNA from cell cultures and melanoma tissues was isolated with TRIzol (Life Technologies) or peqGOLD TriFast (Peqlab) as per manufacturer’s protocol. RNA quality and integrity were assessed using a Bioanalyzer 2100 (Agilent Technologies).

0.5 to 2 μg of RNA was reverse-transcribed with random hexamers (Promega) and 200 U SuperScript II (Life Technologies), using the following protocol: 10 min at 25 °C, 60 min at 42 °C and 15 min at 70 °C. No-reverse-transcriptase controls were included to check for genomic DNA contamination. cDNA relative quantity was then assessed by quantitative PCR using the SYBR Green detection method. Gene-specific primers: Table S6. For each sample, normalized relative quantities were computed using the ΔΔCt method^36^.

### Protein extraction, SDS-PAGE and western blots

Proteins were extracted and separated on SDS-PAGE as described in^7^. They were transferred onto PVDF membranes (Fisher Scientific) using a semi-dry transfer system (Bio-Rad). Membranes were blocked with 5% BSA or 5% nonfat milk in TBST (0.05 or 0.1% Tween-20 in TBS, pH 7.4) for 1 h at room temperature and incubated overnight at 4 °C with primary antibodies diluted in blocking buffer: anti-vinculin (1/10,000; Abcam Cat# ab129002, RRID:AB_11144129) ; anti-GAPDH (1:10,000;Cell Signaling Technology Cat# 2118, RRID:AB_561053); anti-TXNIP (1:1000; Santa Cruz Biotechnology Cat# sc-67134, RRID:AB_2210089 in all figures except Fig. 2A,B; Cell Signaling Technology Cat# 14715, RRID:AB_2714178 in Fig. 2A,B), anti-integrin alpha-11 (1:500; Abcam Cat# ab107858, RRID:AB_10888305), anti-integrin beta-3 (1:2000; Abcam Cat# ab75872, RRID:AB_2249317), anti-MMP-1 (1:500; Millipore Cat# IM35, RRID:AB_2282006), anti-MMP-14 (MT-MMP1; 1:500; Santa Cruz Biotechnology Cat# sc-30074, RRID:AB_2250767), anti-TIMP-2 (1:500, Santa Cruz Biotechnology Cat# sc-6835, RRID:AB_2204951) and anti-collagen alpha3(VI) (1:500; Santa Cruz Biotechnology Cat# sc-47764, RRID:AB_2083110). Membranes were then incubated for 1 h at room temperature with a horseradish peroxidase-conjugated goat anti-rabbit or -mouse secondary antibody (both 1:20,000; Promega Cat# W4011, RRID:AB_430833 and Promega Cat# W4021, RRID:AB_430834) in TBST with 10% blocking buffer. Western blots were revealed using enhanced chemiluminescence and imaged using a Fusion FX7 system (Vilber Lourmat). Densitometric analyses were done with BIO-1D (Vilber Lourmat).

### RNA-seq experiments

For identification of PPARγ-regulated genes, A375 cells were treated with the PPARγ agonist Rosiglitazone (RGZ), with the PPARγ antagonist T0070907 or a combination of both as described in^7^. For identification of TXNIP-regulated genes, A375 were transfected with siTXNIP or siControl. Three independent TXNIP silencing experiments were performed. In each of these 3 experiments, RNA samples from technical replicates of the same condition were pooled. RNA-seq experiments were conducted at the Lausanne Genomic Technologies Facility (Lausanne, Switzerland) according to an in-house pipeline, as described in^7^.

### Microarray dataset analysis

The analyses of two melanoma microarray datasets (GSE46517 and GSE3189; Gene Expression Omnibus database; http://www.ncbi.nlm.nih.gov/geo, accessed on January 19^th^ 2017) are described in^7^.

### RNA expression data analyses

Pre-normalized gene expression data (RPKM) as well as RECIST (Response Evaluation Criteria in Solid Tumors) scores were obtained from Zhang et al. 2016 (EGA ID: EGAS00001000992). Samples were grouped according to their stages: Pre-treatment (stage A), On-treatment (stage B) and Treatment-resistant (stage C). TXNIP (ENSG00000117289) expression values where studied in the tumor stages using the analysis of variance method and compared using a Tukey test. TXNIP expression values were correlated to RECIST scores and other gene expression values (CyclinB1 ENSG00000134057; CyclinD ENSG00000110092; and pRb ENSG00000139687) using linear models.

### In vivo experiments

All experiments involving animals were approved by the Veterinary Office of the Canton Vaud (Switzerland) in accordance with the Federal Swiss Veterinary Office Guidelines and conform to the Directive 2010/63/EU.

Subcutaneous tumor assay was performed as described in^7^.

Metastatic assay: A375-shScr or A375-shTXNIP cells were injected (5 × 10^6^ cells) intravenously into 8 to 10-week-old female NSG mice (IMSR Cat# JAX:001303, RRID:IMSR_JA:001303). Animals were euthanized 3 weeks after intravenous injection. Images of whole lung were acquired using a Leica M205-FA stereo microscope. mCherry fluorescent signal was quantified using ImageJ, by quantifying the intensity of the red fluorescence signal normalized to the surface of the lung. The lungs were processed for total RNA extraction and quantification of the relative expression levels of the human genes *B2M*, *MITF* and *MART* over the murine house-keeping genes *Eef1a1* and *Rsp9* to evaluate the relative amount of human A375-shScr or A375-shTXNIP cells in the murine lungs.

### Statistical analyses

Quantitative RT-qPCR results were analyzed with t tests (paired where appropriate) performed on log-transformed normalized relative quantities. Percent values were converted to fractions and log-transformed before statistical testing. Cell monolayer scraping assays were analyzed by fitting linear models relating the fraction of wound closure to time (both variables were square root-transformed); the slopes obtained for A375-shTXNIP and A375-shScr cells (which quantify cell migration speed) were compared using a F test, the null hypothesis being that they were equal. RNA-seq and microarray data were analyzed as described. *P*-values < 0.05 were considered significant. All statistical analyses were done with GraphPad Prism (RRID:SCR_002798 version #6).

### Equipment and settings

Western blots were imaged using Fusion FX7 system (Vilber Lourmat). Images were saved in Compatibility Plus 16 Bit Tagged Image File Format (*.TIF) without further modification. Densitometric analyses were perfomed with BIO-1D (Vilber Lourmat). Images for visual quantification of fluorescently labelled tumors in whole mouse lungs were acquired using Leica M205-FA stereo microscope.

### Significance

Metastatic dissemination is the major cause of cancer deaths. Getting a better understanding of how malignant cell spread to distant organs thus remains an important issue. This study contributes to this critical objective by revealing a novel role for TXNIP in regulating the expression of proteins key to metastasis formation. Our data strongly suggests that the decrease in *TXNIP* that we describe in advanced melanoma biopsies favors melanoma cell seeding into the lung. Moreover, by identifying *TXNIP* as a PPARγ target, we improve our knowledge of the poorly characterized molecular actors involved in the functions of PPARγ in melanoma.

## Supplementary Information

Supplementary Information.

## Data Availability

RNA-seq experiments: RNAseq transcriptomic analysis of A375 human metastatic melanoma cells treated with the PPARγ agonist RGZ, with the PPARγ antagonist T0070907 or a combination of both is described in^7^ (Primary data accession number: GSE115221). RNAseq transcriptomic analysis of A375 transfected with siTXNIP or siControl was submitted in excel format.

## Abbreviations

BMN: Benign melanocytic nevus
ECM: Extracellular matrix
GO: Gene ontology
MM: Metastatic melanoma
PM: Primary melanoma
ROS: Reactive oxygen species
TXNIP: Thioredoxin-interacting protein
