# Supplementary material for: Low expression of the PPARγ-regulated gene thioredoxin-interacting protein accompanies human melanoma progression and promotes experimental lung metastases
Source: Sci Rep. 2021 Apr 12;11:7847. doi: 10.1038/s41598-021-86329-5 (PMC8042115; doi:10.1038/s41598-021-86329-5)
Supplement: Supplementary file 1 — Supplementary Information. [file 41598_2021_86329_MOESM1_ESM.pdf]

LOW EXPRESSION OF THE PPARγ-REGULATED GENE THIOREDOXIN-INTERACTING PROTEIN ACCOMPANIES HUMAN MELANOMA PROGRESSION AND PROMOTES EXPERIMENTAL LUNG METASTASES

Patrick Meylan[1], Christine Pich[1, 2], Carine Winkler[1], Stefanie Ginster[1], Lionel Mury[1], Marie Sgandurra[1,3], René Dreos[1], Dennie Tompers Frederick[4], Marc Hammond[4], Genevieve Marie Boland[4], Liliane Michalik[1]*

[1]Center for Integrative Genomics, Faculty of Biology and Medicine, University of Lausanne, Lausanne, Switzerland

[2]Current affiliation: Department of Dermatology and Venereology, University Hospital of Lausanne, Centre Hospitalier Universitaire Vaudois, Lausanne, Switzerland

[3]Current affiliation: R&D Philip Morris Products S.A, Neuchâtel, Switzerland

[4]Division of Surgical Oncology, Massachusetts General Hospital, Boston, Massachusetts, USA

*Corresponding author:
Prof. Liliane Michalik
Center for Integrative Genomics
University of Lausanne Sorge
CH-1015 Lausanne
Switzerland
Fax: +41 21 692 39 55
liliane.michalik@unil.ch

SUPPLEMENTARY INFORMATIONS

SUPPLEMENTARY METHODS

Measurement of intracellular ROS levels

A375-shScr and A375-shTXNIP cells were treated for 1 h with increasing concentrations of H_2O_2 (Sigma-Aldrich). Intracellular ROS content was measured using either the microplate-based OxiSelect™ Intracellular ROS assay kit (Cell Biolabs) or the flow cytometry-based CellROX® assay (Life Technologies) as per manufacturer's protocol. Microplates were read with a Safire2 multidetection microplate reader (Tecan). For flow cytometry, cells were analyzed on a BD LSR-II flow cytometer equipped with FACSDiva software (both from BD Biosciences). Data were analyzed using FlowJO (FlowJo, RRID:SCR_008520)). ROS levels were measured in at least 10000 cells per sample.

Flow cytometry analysis of cell cycle

A375-shScr, A375-shTXNIP C8161-shTXNIP, and C8161-shScr cells were collected with Hank's based cell dissociation buffer (Life Technologies) and washed in PBS.

Cell cycle was analyzed by flow cytometry using the Click-iT® EdU Alexa Fluor® 647 assay kit (Life Technologies) combined with DAPI staining, as per manufacturer's instructions. Briefly, A375 cells plated onto 60-mm dishes were incubated for 30 min at 37°C with 10 μM EdU, detached with trypsin and washed with DPBS (PBS with 1 mM $CaCl_2$ and 0.5 mM $MgCl_2$). Cells were then fixed with 4% paraformaldehyde in PBS for 15 min at room temperature, washed and incubated for 30 min at room temperature in Click-iT cocktail. After a final wash, cells were incubated for 15 min at room temperature in a 2.5 μg/ml DAPI (Sigma-Aldrich) solution made in DPBS plus 0.1% NP-40 (AppliChem). Cells were stored for a maximum of 1h at 4°C before flow cytometry.

Transduction of the C8161 human melanoma cell line with TXNIP ORF

60,000 C8161 cells were seeded into a 66mm dish in DMEM with 10%FBS and 5%PenStrep. The medium was changed 24h later and 4ul of polybrene were added to 5ml of medium. Cells were transduced with 15ul viral particles (Genecopeia; Negative Ctrl: LPP-NEG-Lv125-A00; TXNIP ORF: LPP-M0226-Lv125-A00) for 16h, then cultured in fresh medium for 48h. Transduced cells were then selected with 1.25μg/ml Puromycin.

SUPPLEMENTARY TABLES

Supplementary Table S1. Enrichment of protein classes upon TXNIP knockdown in A375 cells

**Protein class**	**Enrichment ratio**	**P-value**
Ligands	1.888	5.5e-05
Receptors	1.734	1.7e-09
Kinases	1.49	6.3e-03
Proteases	1.303	7.4e-02
Phosphatases	1.281	2.2e-01
Transcription factors	1.138	1.9e-01
Enzymes	1.003	5.0e-01
Other	0.8834	1.6e-10

Supplementary Table S2. Top 20 significantly deregulated GO localizations upon TXNIP knockdown in A375 cells

GO localization	Total nb. of genes in the pathway	Nb. of SDG[1] in the pathway	Adjusted P-value
Cell periphery	5773	452	2.1e-31
Plasma membrane	5650	441	2.9e-30
Plasma membrane part	2658	246	4.5e-24
Intrinsic component of plasma membrane	1594	162	7.5e-19
Integral component of plasma membrane	1520	151	1.4e-16
Extracellular region part	4173	297	1.4e-12
Extracellular region	5304	357	2.5e-12
Membrane	10655	624	4.0e-12
Vesicle	3933	275	1.1e-10
Membrane part	7895	477	8.1e-10
Extracellular matrix	489	60	8.1e-10
Membrane-bounded vesicle	3797	263	8.8e-10
Extracellular space	1549	132	9.7e-10
Cell surface	842	84	3.5e-9
Cell junction	1195	107	5.0e-9
Intrinsic component of membrane	6793	415	8.9e-9
Proteinaceous extracellular matrix	399	50	1.3e-8
Endomembrane system	4017	268	1.9e-8
Cell projection	1905	147	5.2e-8
Integral component of membrane	6636	402	5.5e-8

[1] *SDG*, significantly deregulated genes

Supplementary Table S3. Top 20 significantly deregulated GO molecular functions upon TXNIP knockdown in A375 cells

GO molecular function	Total nb. of genes in the pathway	Nb. of SDG[2] in the pathway	Adjusted P-value
Protein binding	10424	653	2.4e-20
Binding	15196	821	1.1e-9
Receptor binding	1719	137	1.7e-6
Growth factor binding	143	26	2.1e-6
C5L2 anaphylatoxin chemotactic receptor binding	6	6	4.3e-6
Substance P receptor binding	5	5	6.3e-5
Neurokinin receptor binding	5	5	6.3e-5
Cell adhesion molecule binding	198	28	7.3e-5
Platelet-derived growth factor binding	12	7	7.3e-5
C5a anaphylatoxin chemotactic receptor binding	9	6	1.5e-4
Calcium ion binding	827	72	1.5e-4
Platelet-derived growth factor receptor binding	16	7	6.7e-4
Transmembrane receptor protein kinase activity	104	17	1.3e-3
Glycosaminoglycan binding	232	27	2.8e-3
Protein dimerization activity	1299	95	3.8e-3
Metalloendopeptidase activity	126	18	3.8e-3
Heparin binding	176	22	4.3e-3
Integrin binding	117	17	4.5e-3
Extracellular matrix structural constituent conferring tensile strength	6	4	6.7e-3
Transmembrane receptor protein tyrosine kinase activity	78	13	7.4e-3

[2] *SDG*, significantly deregulated genes

Supplementary Table S4. Top 5 significantly deregulated GeneGO pathway maps upon TXNIP knockdown in A375 cells

Pathway name	Total nb. of genes in the pathway	Nb. of SDG[3] in the pathway	Adjusted P-value	SDG in the pathway (fold change of si-TXNIP vs. si-ctrl)
Development_Oligodendrocyte differentiation from adult stem cells	51	13	7.2e-5	↑: PDGFRA (5.8), BMP4 (3.5), EDNRA (3.4), SLCO1A2 (2.5), SMAD7 (2.5), BMP2 (2.5), FGFR3 (2.5), SLC16A2 (2.4), EDNRB (2.3), MAPK13 (2.1), BMPR2 (2.0)<br>↓: EDN1 (-2.8), PDGFB (-2.2)
Cell adhesion_ECM remodeling	52	12	3.6e-4	↑: MMP9 (6.9), COL2A1 (6.4), PLAU (4.1), MMP14 (4.0), CXCL8 (3.5), COL4A1 (2.8), VCAN (2.8), FN1 (2.4), MMP13 (2.4), MMP16 (2.2), MMP1 (2.0), TIMP2 (2.0)
Development_Regulation of epithelial-to-mesenchymal transition (EMT)	64	13	4.0e-4	↑: MMP9 (6.9), PDGFRA (5.8), WNT7B (4.6), JAG1 (3.7), HEY1 (3.5), EDNRA (3.4), FN1 (2.4), PDGFRB (2.4), SKIL (2.2)<br>↓: CLDN1 (-8.0), EDN1 (-2.8), MET (-2.8), PDGFB (-2.2)
Normal and pathological TGF-β-mediated regulation of cell proliferation	33	8	9.4e-4	↑: AXIN2 (7.0), PDGFRA (5.8), ITGB3 (2.6), PDGFRB (2.4), MAPK13 (2.1)<br>↓: MYC (-3.1), PDGFB (-2.2), RAC1 (-2.2)
Development_WNT signaling pathway. Part 2	53	10	7.5e-3	↑: AXIN2 (7.0), WNT7B (4.6), BMP4 (3.5), ENC1 (2.8), NRCAM (2.7), TCF (2.6), APC (2.1)<br>↓: CLDN1 (-8.0), DKK1 (-4.5), MYC (-3.1)

[3] *SDG*, significantly deregulated genes

Supplementary Table S5. Top 20 significantly deregulated KEGG pathways upon TXNIP knockdown in A375 cells

Pathway name	Total nb. of genes in the pathway	Nb. of SDG[4] in the pathway	Adjusted P-value
Pathways in cancer	326	40	1.6e-14
ECM-receptor interaction	85	20	1.3e-12
Focal adhesion	200	28	7.4e-12
Regulation of actin cytoskeleton	213	25	5.3e-9
Arrhythmogenic right ventricular cardiomyopathy	74	15	9.0e-9
Axon guidance	129	19	1.1e-8
Cytokine-cytokine receptor interaction	265	25	2.8e-7
MAPK signaling pathway	268	24	1.3e-6
Metabolic pathways	1130	59	1.4e-6
Cell adhesion molecules	133	16	2.8e-6
Protein digestion and absorption	81	12	8.7e-6
Basal cell carcinoma	55	10	9.5e-6
Hypertrophic cardiomyopathy	83	12	9.6e-6
Hedgehog signaling pathway	56	10	9.7e-6
Wnt signaling pathway	150	16	9.7e-6
Dilated cardiomyopathy	90	12	1.9e-5
Rheumatoid arthritis	91	12	2.0e-5
Endocytosis	201	18	2.3e-5
Phagosome	153	15	4.7e-5
TGF-beta signaling pathway	84	11	4.7e-5

[4] *SDG*, significantly deregulated genes

Supplementary Table S6. Primer sequences

Gene symbol	Forward primer (5'-3')	Reverse primer (5'-3')
B2M[5]	AGGCTATCCAGCGTACTCCAAAGA	TCGGATGGATGAAACCCAGACACA
CCNB1	GATTGGAGAGGTTGATGTCGAGCAA	GATTGGAGAGGTTGATGTCGAGCAA
CDKN2A	CCC CCA CTA CCG TAA ATG TCC	TCC AAC ACA GTG AAA AGG CAG
COL4A1	AACCAGGACCCAGAGGCAAA	AACCAGGACCCAGAGGCAAA
COL6A1	CAGATTACACCTGCCCCATCAC	CAGATTACACCTGCCCCATCAC
COL6A2	GGTGCTGCGACTGTGAGAAG	GGTGCTGCGACTGTGAGAAG
COL6A3	CTACCGAGCCCAGGTGTTG	CTACCGAGCCCAGGTGTTG
FN1	AAGCCCTACCAAGGCTGGAT	AAGCCCTACCAAGGCTGGAT
GAPDH[5]	CATCCATGACAACTTTGGTATCGT	CCATCACGCCACAGTTTCC
HPRT1[5]	CTCCTCCTCTGCTCCGCCAC	CATCACTAATCACGACGCCAGGG
ITGA1	CACCAACCCAAATGGAGGATTTC	CACCAACCCAAATGGAGGATTTC
ITGA11	ACGCTGTGATTCTGTGGTCC	ACGCTGTGATTCTGTGGTCC
ITGA5	GATCAACCTGGTTCAGGGGC	GATCAACCTGGTTCAGGGGC
ITGA6	CACCAACCCAAATGGAGGATTTC	CACCAACCCAAATGGAGGATTTC
ITGB1	CAGTGTGTTTGTAGGAAGAGGG	CAAACACCATTTCCTCCACAAATTAA G
ITGB2	ATCATCCCCAAGTCAGCCG	ATCATCCCCAAGTCAGCCG
ITGB3	AACCCCTGCTATGATATGAAGACC	AACCCCTGCTATGATATGAAGACC
ITGB5	CGGCTCGCAGGTCTCAA	CGGCTCGCAGGTCTCAA
ITGB8	AAGCCTATCAGAAGCTCATTTCAGA AG	AAGCCTATCAGAAGCTCATTTCAGAA G
MMP1	TGGACCAACAATTTCAGAGAGTACA	TGGACCAACAATTTCAGAGAGTACA
MMP13	AACCTACAGAATTGTGAATTACACCC	AACCTACAGAATTGTGAATTACACCC
MMP14	CATCCAGGGTCTCAAATGGCA	CATCCAGGGTCTCAAATGGCA
MMP16	GGAAGACGGTTGGATTTCGTG	GGAAGACGGTTGGATTTCGTG
MMP2	GGCTGGTCAGTGGCTTGGGGTA	AGATCTTCTTCTTCAAGGACCGGTT
MMP8	CCCTTCCAACTGGTATACAGGC	CCCTTCCAACTGGTATACAGGC
PCNA	ATGGGCGTGAACCTCACCAGTATG	ATGGGCGTGAACCTCACCAGTATG
TBP[5]	GCCCGAAACGCCGAATATA	CGTGGCTCTCTTATCCTCATGA
TFRC[5]	CGGTCATCAGGATTGCCTAATATAC	CATTCTTGCTTTCTGAGGTTACCA
TIMP2	QT00017759 (Qiagen)	QT00017759 (Qiagen)
TXNIP	TACCCCTGATTTAATGGCACC	CAATTCGAGCAGAGACAGACAC
USP16[5]	TGGGCTCTGTCGCCGTGGATTG	TGTCCGTTTCTTTCCCATGTTGGCAC
YWHAZ[5]	TTGAAAATGAAAGGAGATTACTACC GTTA	ACTGATCGACAATCCCTTTCTTG
Rps9[6]	GAC CAG GAG CTA AAG TTG ATT GGA	TCT TGG CCA GGG TAA ACT TGA
Eef1a1[6]	TCA TGT CAC GAA CAG CAA AGC	CCT GGC AAG CCC ATG TGT

[5] housekeeping genes
[6] primers for mouse cDNA

SUPPLEMENTARY FIGURE LEGENDS

Supplementary Figure 1

(A) Expression of *TXNIP* mRNA (log2 scale) in the same microarray dataset as in Fig. 2B (GSE46517) comparing 7 benign melanocytic nevi (BMN), 8 primary melanomas (PM) and 57 metastatic melanomas (MM) stratified by tumor stage according to the AJCC staging system (8th edition, 2014). Open circles represent individual samples, lines represent means. **(B, C)** qRT-PCR analysis of *TXNIP* (B) and *PCNA* (C) mRNA expression level in patient melanoma metastases excised before treatment (Pre-treatment), early after MAP kinase targeted therapy initiation (On-treatment) or during progression of the disease (Treatment-resistant). Patient clinical data are presented in Table 1. Columns represent means. Color lines represent samples excised longitudinally from individual patients. House-keeping genes: *YWHAZ*, *B2M* and *USP16*. **(D)** Percent of Ki67-labelled melanoma cells in patient melanoma metastases excised before treatment (Pre-treatment) or early after MAP kinase targeted therapy initiation (On-treatment). Patient clinical data are presented in Table 1. Bars represent means. Color lines represent samples excised longitudinally from individual patients. All panels *, $P < 0.05$; **, $P < 0.01$; ***, $P < 0.001$.

Supplementary Figure 2

(A) Left panel: qRT-PCR analysis of *TXNIP* mRNA expression level in polyclonal A375-shScr and A375-shTXNIP cell lines. Each open circle represents one experiment performed with biological triplicates (n=3). Five independent experiments were performed (N = 5). Columns are means independent experiments. House-keeping genes: *YWHAZ* and *USP16*. Statistics:paired Student's t-test. **Right panel:** Western blot analysis of TXNIP protein level in total proteins extracted from A375-shScr and A375-shTXNIP. GAPDH was used as loading control and was run on the same gel. Cropped western blots are shown. Full-length western blots and densitometry are shown in Supplementary Figure 8. **(B)** Representative FACS plot of EdU- and DAPI-labeled A375-shScr (left) and A375-shTXNIP cells (middle); quantification of cell cycle distribution of A375-shScr and A375-shTXNIP cells as quantified by flow cytometry (right). Each open circle represents one experiment performed with biological duplicates (n=2). Three independent experiments were performed (N = 3). Columns are means independent experiments. Statistics: paired Student's t-test. **(C)** qRT-PCR analysis of *CCNB1, CDKN2A* and *PCNA* mRNA expression levels in A375-shScr and A375-shTXNIP cells. Each open circle represents one experiment performed with biological triplicates (n=3). Three independent experiments were performed (N = 3). Columns are means of three independent independent experiments. House-keeping genes: *YWHAZ* and *USP16*. Statistics: paired Student's t-test. **(D)** Western blot analysis of total and cleaved Caspase-3 (Casp-3) protein level in total proteins extracted from A375-shScr and A375-shTXNIP (left). Total proteins extracted from immortalized keratinocytes (HaCaT) exposed to UVB (30 and 100 mJ/cm^2) were used as a positive control for caspase-3 activation (right). Actin was used as a loading control and run on the same gel. Cropped western blots are shown. Full-length western blots and densitometry are shown in Supplementary Figure 8. **(E)** qRT-PCR analysis of *TXNIP* mRNA expression level in A375 cells transfected with control (siScr) or TXNIP (siTXNIP) siRNA. Each open circle represents one experiment performed with biological triplicates (n=3). Three independent experiments were performed (N = 3). Columns are means of three independent experiments. House-keeping genes: *YWHAZ* and *USP16*. Statistics: paired Student's t-test. All panel: *, $P < 0.05$

Supplementary Figure 3.

(A, B, C, D) Western blot analyses of integrin alpha-11; collagen alpha-3(VI); matrix metalloproteinase-1 (MMP-1) and matrix metalloproteinase-14 (MMP-14) protein levels in total proteins extracted from A375-shScr and A375-shTXNIP cells; vinculin (Vinc) or GAPDH were used as loading controls and were run on the same gel as the protein of interest. Cropped western blots are shown. Full-length western blots and densitometry are shown in Supplementary Figure 9. (E) Western blot analyses of TXNIP in total proteins extracted from C8161-shScr and C8161-shTXNIP cells; actin was used as loading controls and was run on the same gel. Cropped western blots are shown. Full-length western blots and densitometry are shown in Supplementary Figure 9. (F) Control C8161 (C8161 Ctl; short non-coding stuffer) and C8161-TXNIP ORF cell monolayer scraping assays. Grey circles/dotted line and black squares/plain line each represent the mean of two independent experiments (N = 3); each independent experiment includes biological triplicates (n=3). Data are expressed as means ± SD. (G) Intracellular reactive oxygen species (ROS) content in A375-shScr and A375-shTXNIP cells treated for 1h with increasing concentrations of H_2O_2 (N = 1).

Supplementary Figure 4.
Full-length western blots and densitometry of Figure 3B

Supplementary Figure 5.
Full-length western blots and densitometry of Figure 3D

Supplementary Figure 6.
Full-length western blots and densitometry of Figure 3F

Supplementary Figure 7.
Full-length western blots and densitometry of Figure 4

Supplementary Figure 8.
Full-length western blots and densitometry of Supplementary Figure 2

Supplementary Figure 9.
Full-length western blots and densitometry of Supplementary Figure 3A-D

Supplementary Figure 10.
Full-length western blots and densitometry of Supplementary Figure 3E-F

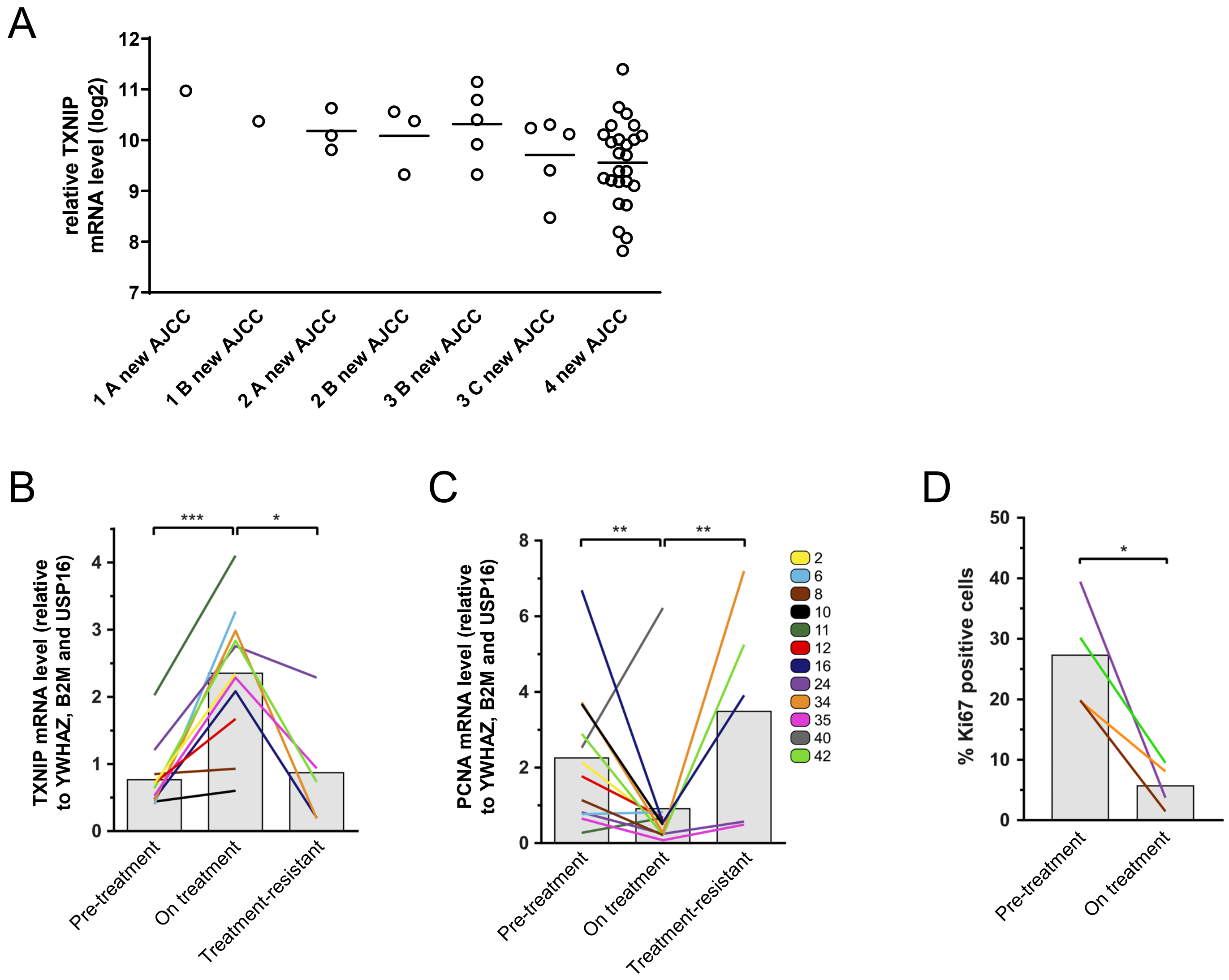

Supplementary Figure 1

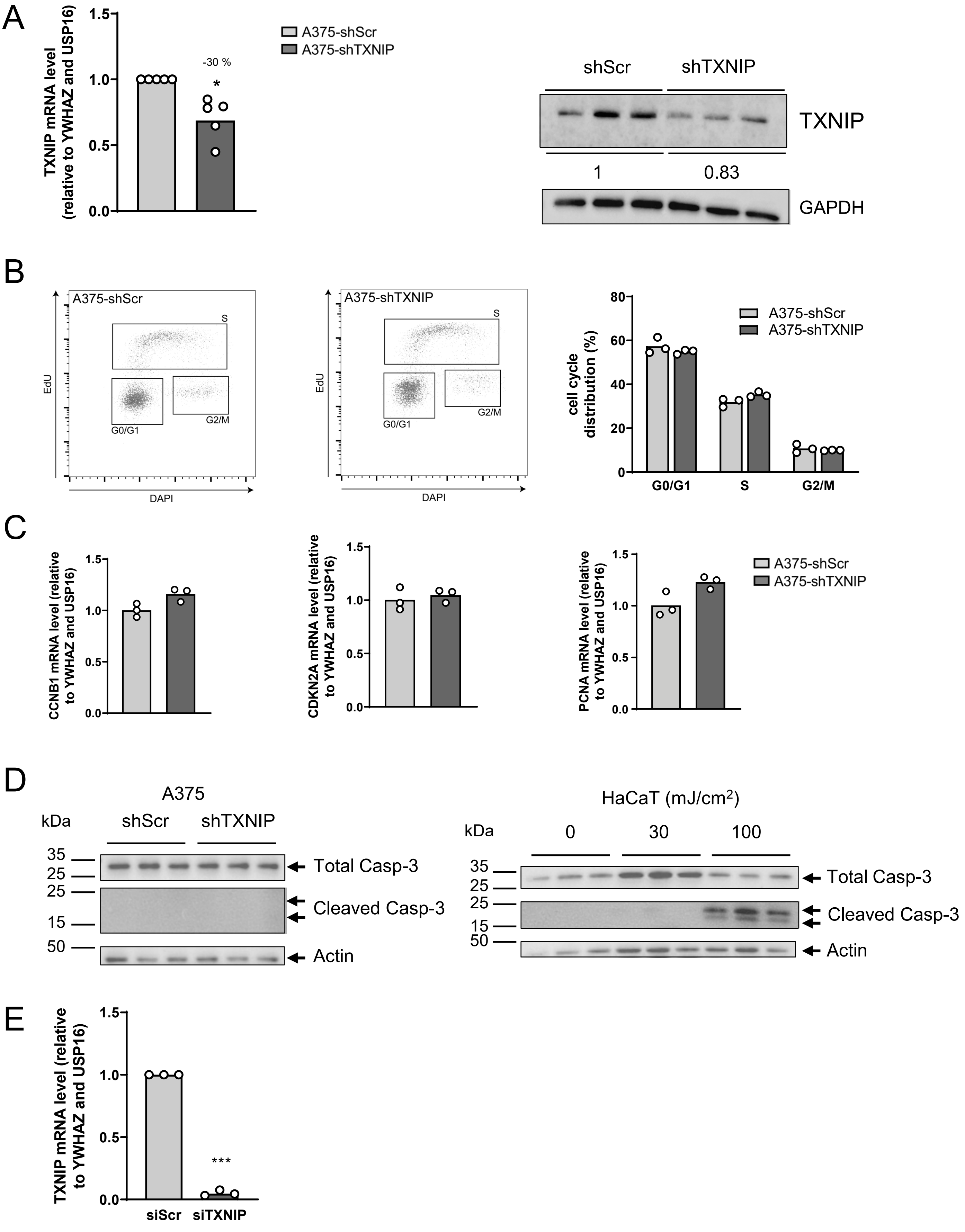

Supplementary Figure 2

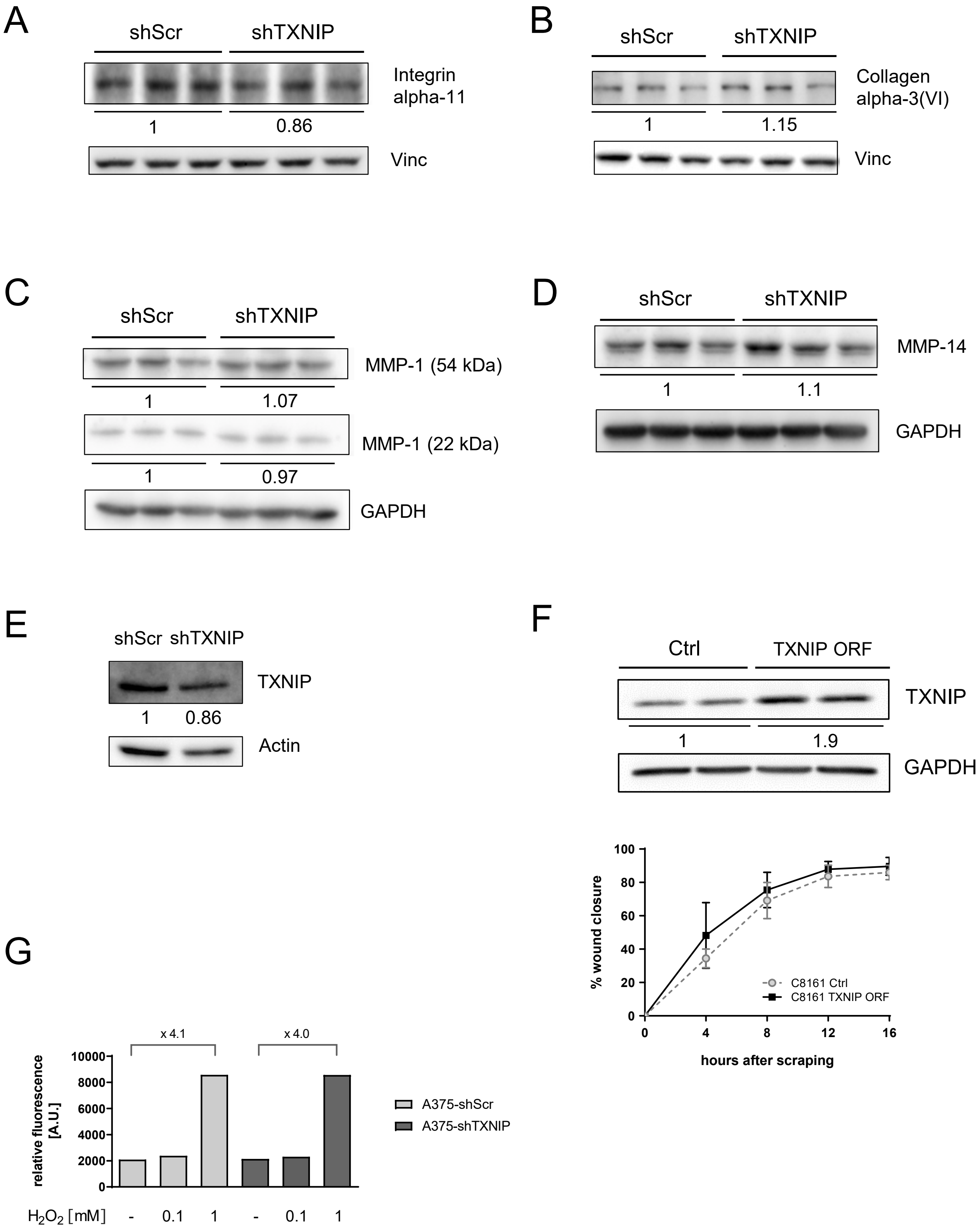

Supplementary Figure 3

Full-length western blots and densitometry of Figure 3B

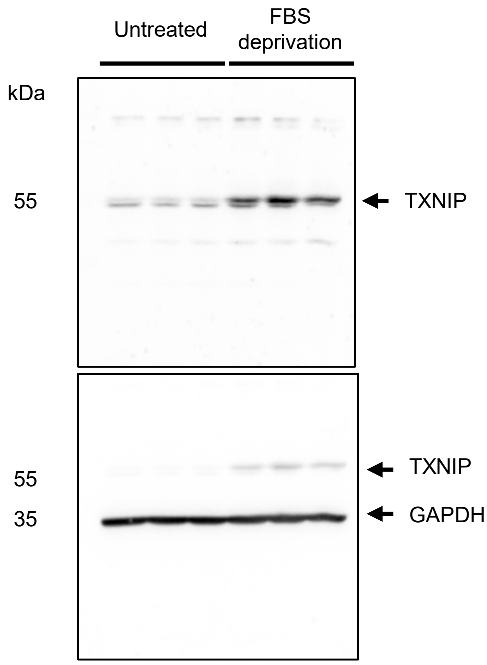

Quantification	Untreated			FBS Deprivation		
TXNIP	9,0	7,5	8,8	23,0	29,3	22,4
GAPDH	20,0	17,2	17,2	15,9	15,4	14,5
TXNIP/GAPDH	0,45	0,44	0,51	1,45	1,90	1,55

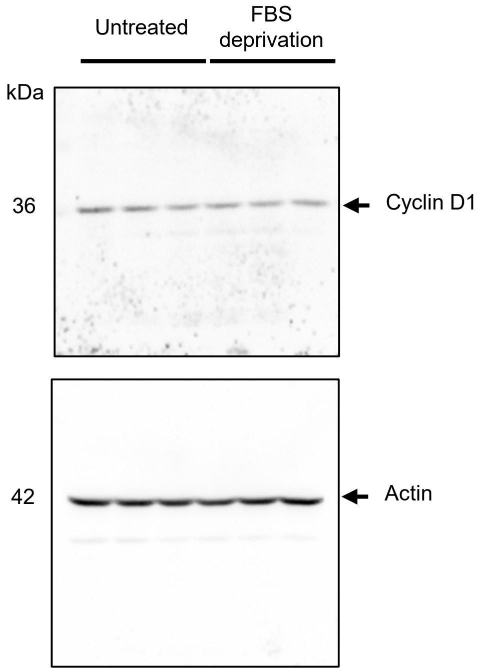

Quantification	Untreated			FBS Deprivation		
CyclinD1	22,5	18,7	13,7	15,5	13,6	15,9
Actin	19,1	16,5	14,7	14,3	16,7	18,6
CyclinD1/Actin	1,18	1,13	0,94	1,08	0,82	0,85

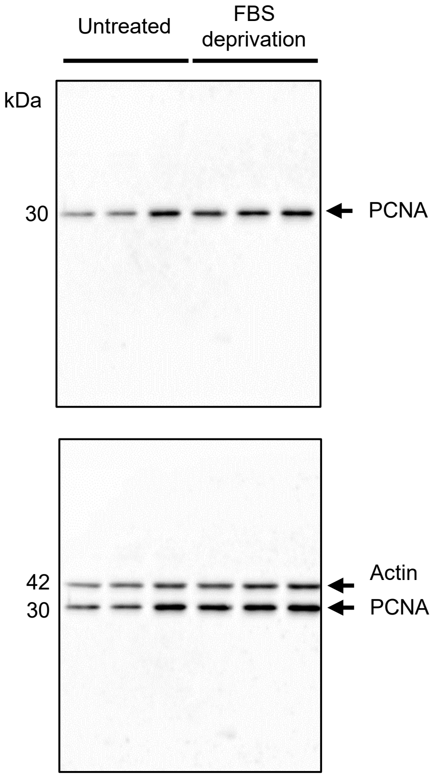

Quantification	Untreated			FBS Deprivation		
PCNA	7,6	8,4	17,7	14,6	15,8	17,6
Actin	7,9	10,4	13,9	14,6	16,1	17,4
Ratio	0,96	0,81	1,27	1,00	0,98	1,02

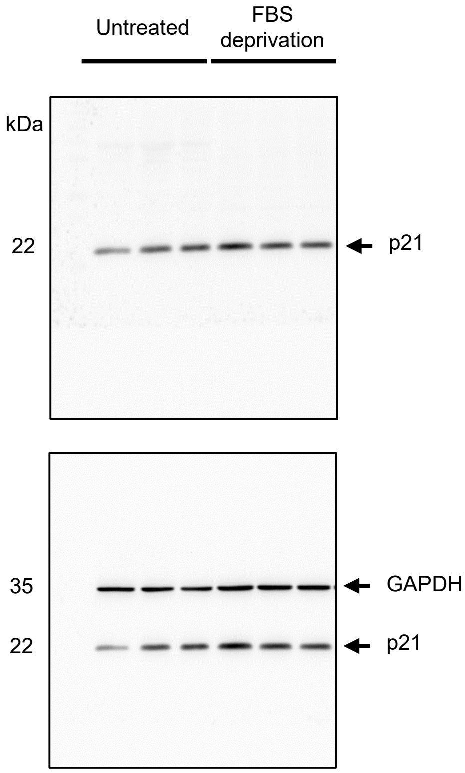

Quantification	Untreated			FBS Deprivation		
p21	6,6	9,5	10,2	14,1	10,8	10,1
GAPDH	10,5	9,3	7,3	11,8	11,3	10,4
Ratio	0,63	1,03	1,40	1,20	0,96	0,97

Supplementary Figure 4

Full-length western blots and densitometry of Figure 3D

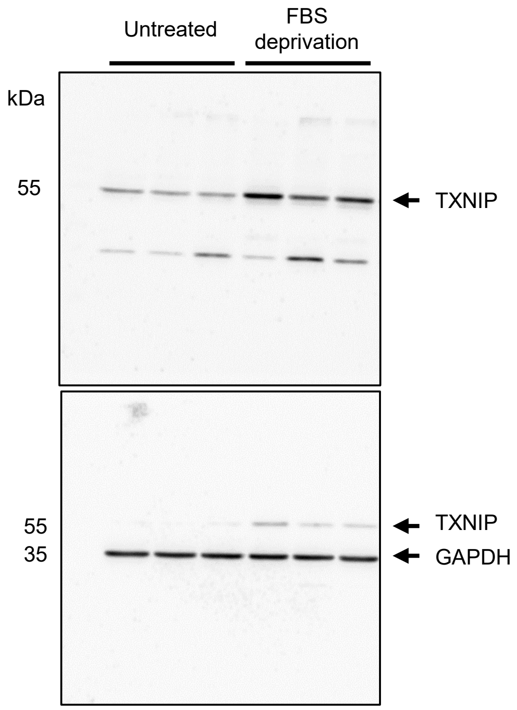

Quantification	Untreated			FBS Deprivation		
TXNIP	5,9	4,7	5,2	17,7	10,5	12,9
GAPDH	11,8	11,3	11,2	11,3	10,9	9,6
Ratio	0,50	0,41	0,46	1,56	0,96	1,35

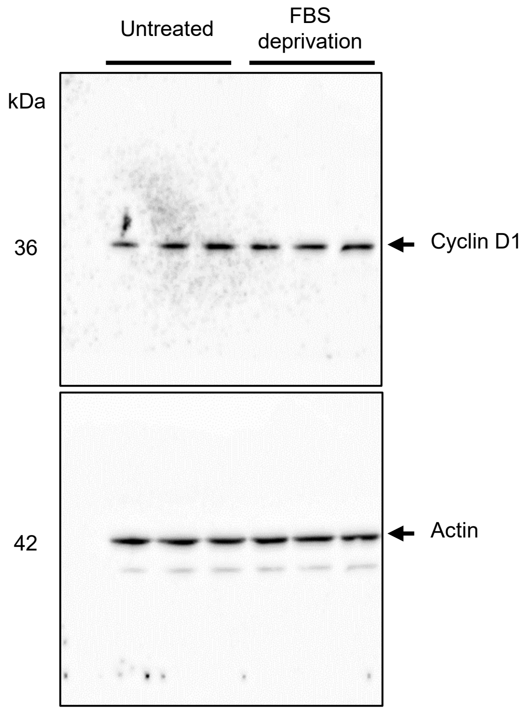

Quantification	Untreated			FBS Deprivation		
CyclinD1	7,4	13,1	15,3	11,1	10,0	11,4
Actin	11,3	11,0	10,0	10,6	10,9	10,1
Ratio	0,65	1,19	1,52	1,05	0,92	1,13

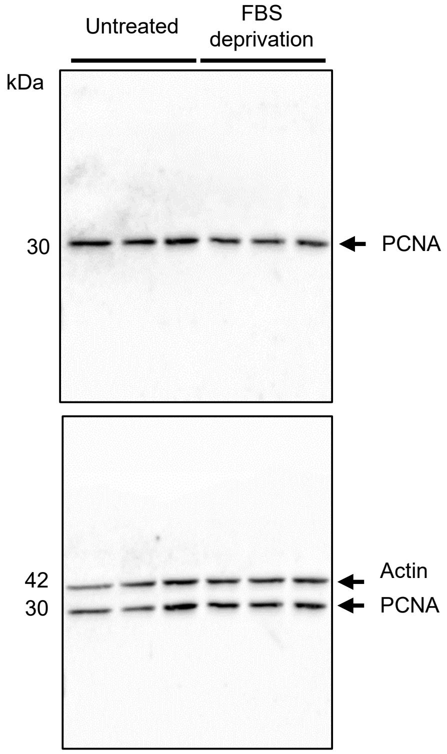

Quantification	Untreated			FBS Deprivation		
PCNA	22,6	14,9	16,6	11,4	11,6	11,5
Actin	11,0	12,8	16,7	13,7	14,0	14,3
Ratio	2,05	1,17	0,99	0,83	0,83	0,81

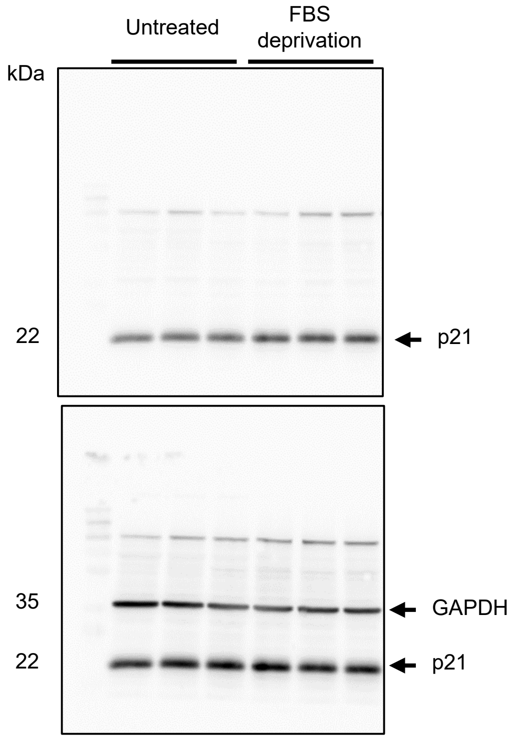

Quantification	Untreated			FBS Deprivation		
p21	7,8	8,7	8,8	10,8	11,5	10,9
GAPDH	17,5	14,2	9,9	9,3	11,4	10,3
Ratio	0,44	0,61	0,89	1,17	1,00	1,07

Supplementary Figure 5

Full-length western blots and densitometry of Figure 3F

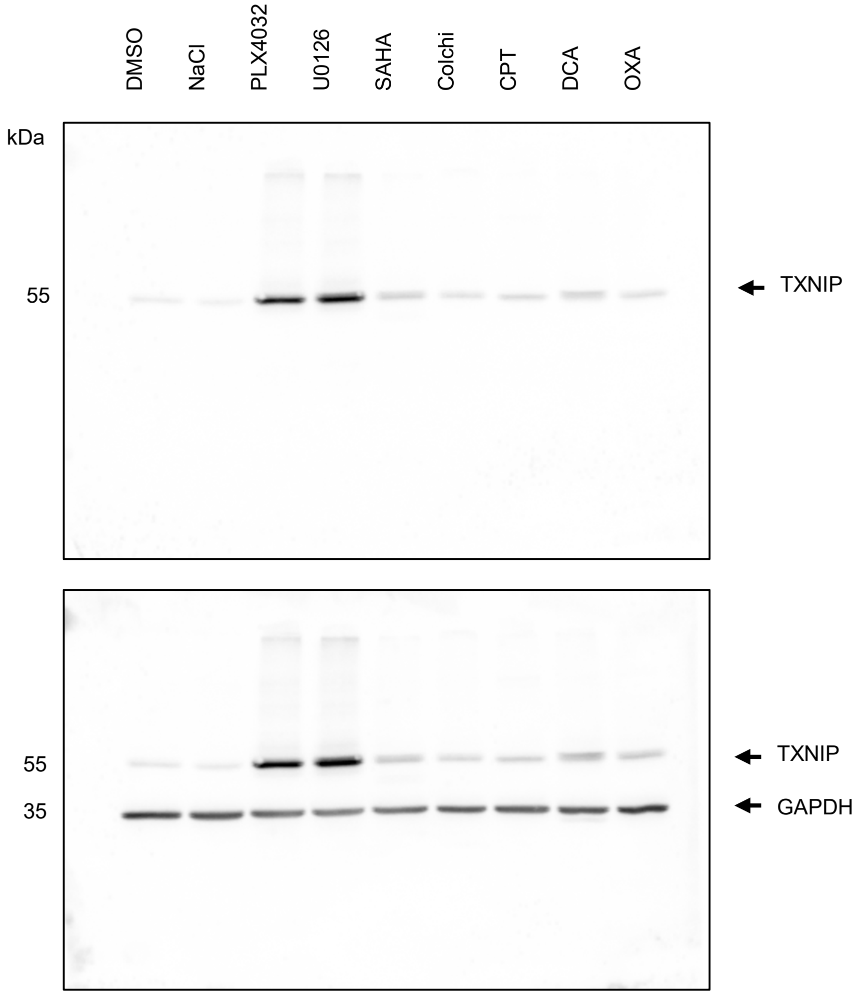

Quantification	DMSO	NaCl	PLX	U0126	CPT	Colchi	SAHA	DCA	OXA
TXNIP	4,806	3,864	35,114	41,421	9,17	5,624	6,564	8,234	5,88
GAPDH	21,509	19,055	15,924	13,382	15,105	15,024	17,405	17,207	19,627
Ratio	0,2234	0,2028	2,2051	3,0953	0,6071	0,3743	0,3771	0,4785	0,2996

Supplementary Figure 6

Full-length western blots and densitometry of Figure 4

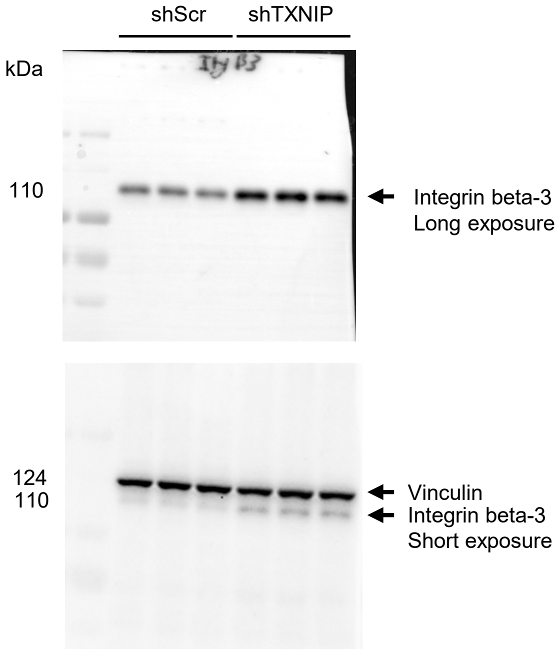

Quantification	sh-scr			sh-TXNIP		
Integrin beta-3	20 039 572	21 209 059	19 479 423	37 234 700	40 261 531	38 830 561
Vinculin	84 648	85 148	85 981	77 202	80 978	76 951
Ratio	236,74	249,08	226,55	482,30	497,19	504,61

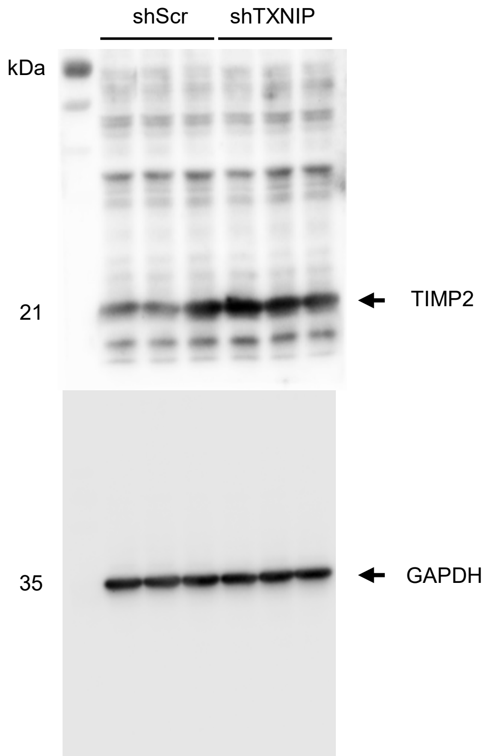

Quantification	sh-scr			sh-TXNIP		
TIMP2	23 272 983	23 037 750	37 737 699	53 215 088	44 961 847	31 586 726
GAPDH	29 740 230	27 799 461	30 652 040	30 963 598	31 945 581	30 015 339
Ratio	0,78	0,83	1,23	1,72	1,41	1,05

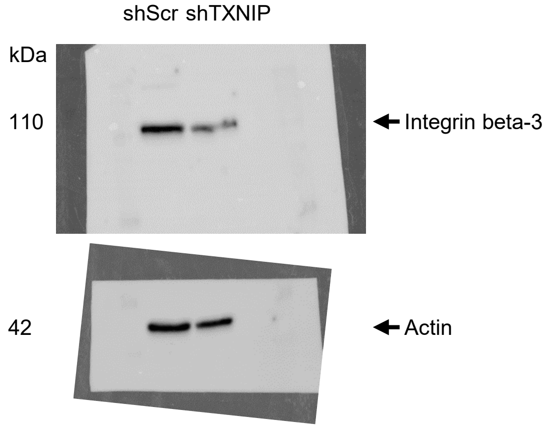

Quantification	sh-scr	sh-TXNIP
Integrin beta-3	64 486 119	47 703 180
Actin	76 042 451	69 222 345
Ratio	0,85	0,69

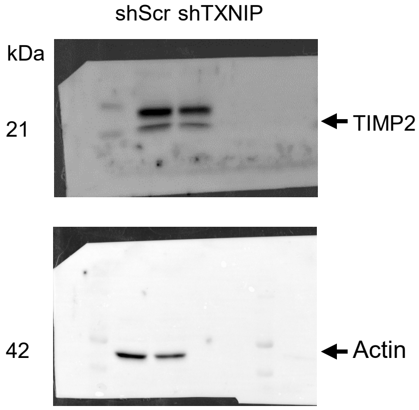

Quantification	sh-scr	sh-TXNIP
TIMP2	51 831 899	47 389 066
Actin	62 866 813	57 455 662
Ratio	0,82	0,82

Supplementary Figure 7

Full-length western blots and densitometry of Supplementary Figure 2

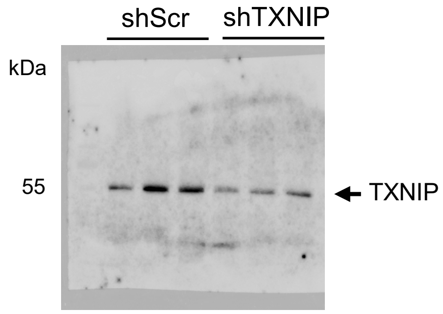

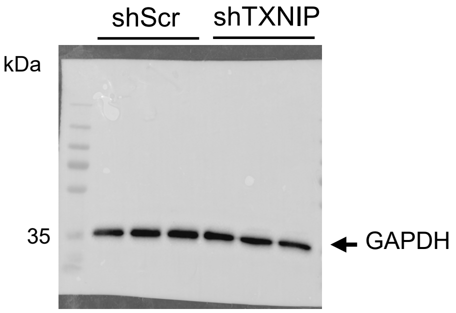

Quantification	sh-scr			sh-TXNIP		
TXNIP	65 896 959	79 811 590	86 141 597	65 860 188	65 536 634	63 668 902
GAPDH	74 443 319	75 101 958	85 215 996	78 953 922	76 682 184	78 973 418
Ratio	0,89	1,06	1,01	0,83	0,85	0,81

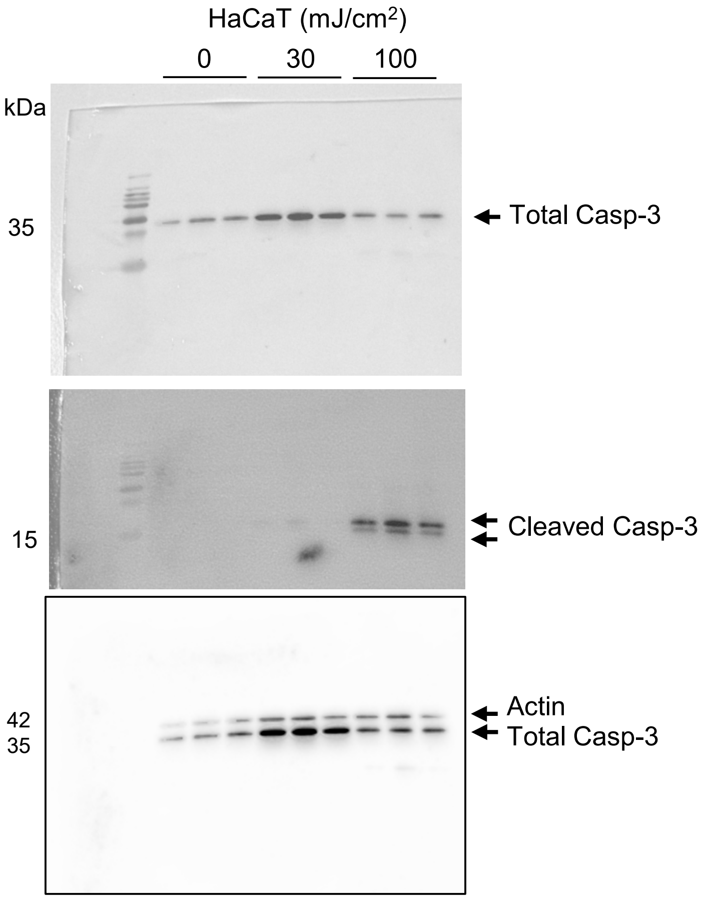

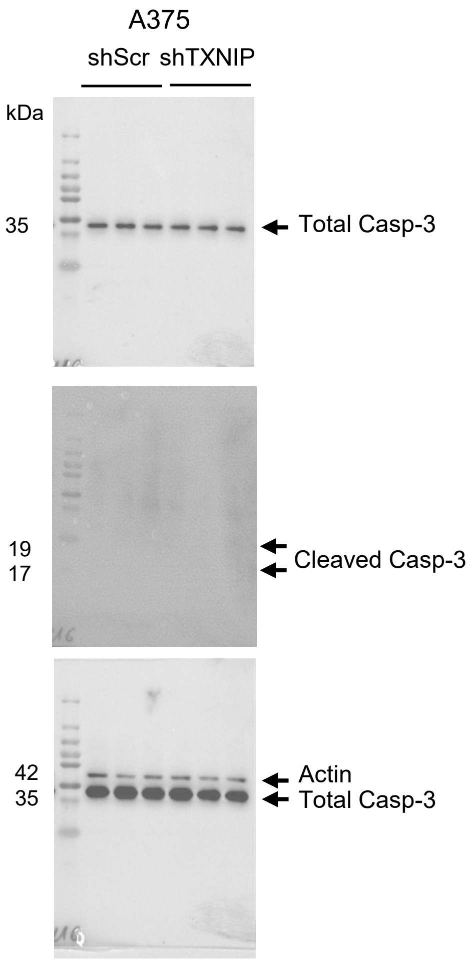

Quantification	0 (mJ/cm2)			30 (mJ/cm2)			100 (mJ/cm2)		
Cleaved Casp-3	5,14	3,63	2,07	1,84	2,98	2,21	20,97	37,53	23,65
Total Casp-3	4,28	9,19	9,12	24,80	29,65	22,97	9,33	7,36	9,80
Actin	6,68	12,13	16,97	22,94	22,45	18,83	18,89	23,80	13,61

Quantification	sh-scr			sh-TXNIP		
Total Casp-3	17,52	18,59	15,57	16,27	16,43	15,62
Actin	20,86	14,46	16,59	17,50	15,14	15,46

Supplementary Figure 8

Full-length western blots and densitometry for Supplementary Figure 3A-D

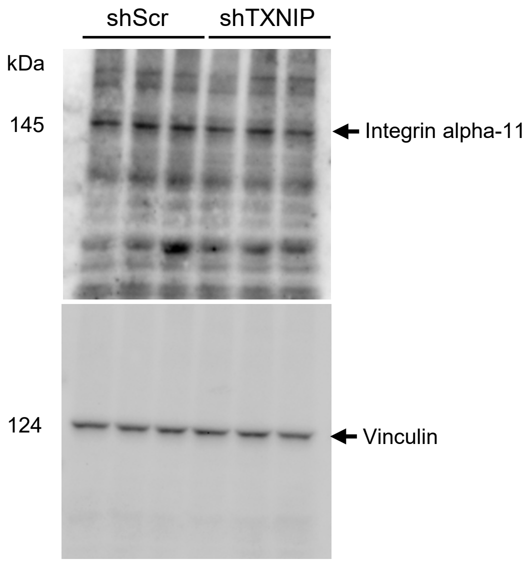

Quantification	sh-scr			sh-TXNIP		
Integrin alph2a-11	12 618 317	16 905 383	15 582 033	13 505 818	15 713 257	10 349 154
Vinculin	9 453 575	9 169 841	8 952 829	9 964 982	9 434 689	8 631 169
Ratio	1,33	1,84	1,74	1,36	1,67	1,20

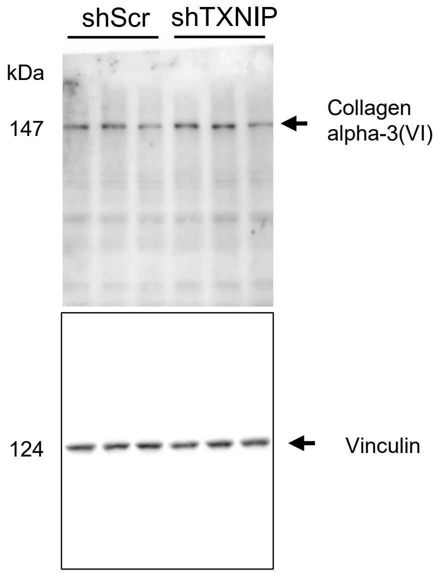

Quantification	sh-scr			sh-TXNIP		
Collagen alpha-3(VI)	11 213 089	10 558 340	7 058 202	11 613 476	11 515 157	8 057 088
Vinculin	13 670 874	11 804 388	9 366 276	9 837 303	11 009 743	11 894 774
Ratio	0,82	0,89	0,75	1,18	1,05	0,68

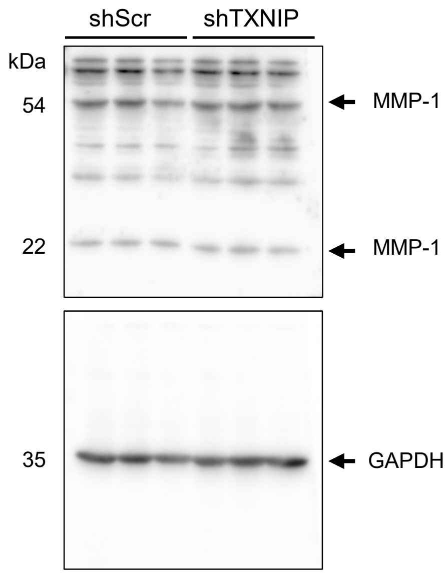

Quantification	sh-scr			sh-TXNIP		
MMP-1 54KDa	15 684 984	17 012 042	7 532 105	13 105 425	16 498 668	12 461 745
GAPDH	19 751 582	16 663 817	14 124 482	14 083 103	18 991 261	18 849 665
Ratio	0,79	1,02	0,53	0,93	0,87	0,66

Quantification	sh-scr			sh-TXNIP		
MMP-1 22KDa	15 731 693	16 799 682	16 301 233	15 072 764	19 872 146	14 156 304
GAPDH	19 751 582	16 663 817	14 124 482	14 083 103	18 991 261	18 849 665
Ratio	0,80	1,01	1,15	1,07	1,05	0,75

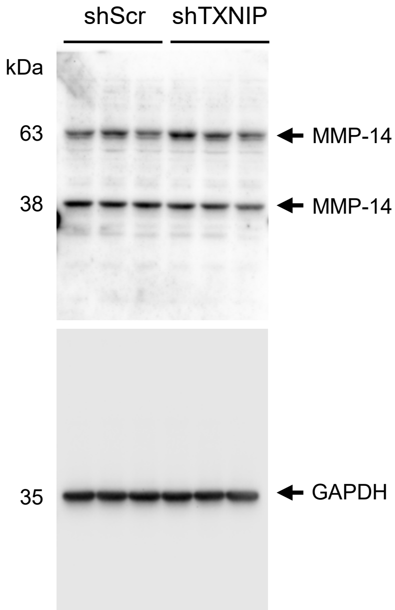

Quantification	sh-scr			sh-TXNIP		
MMP-14 63kDa	27 363 296	31 621 624	24 039 311	34 318 962	27 395 935	23 329 067
GAPDH	31 607 823	31 540 796	31 244 741	30 793 997	30 124 256	26 890 952
Ratio	0,87	1,00	0,77	1,11	0,91	0,87

Supplementary Figure 9

Full-length western blots and densitometry for Supplementary Figure 3E-F

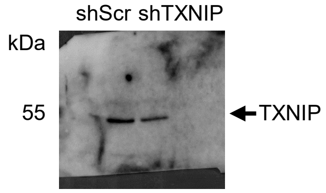

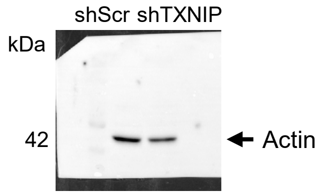

Quantification	sh-scr	sh-TXNIP
TXNIP	58 299 440	53 655 256
Actin	61 856 930	65 945 324
Ratio	0,94	0,81

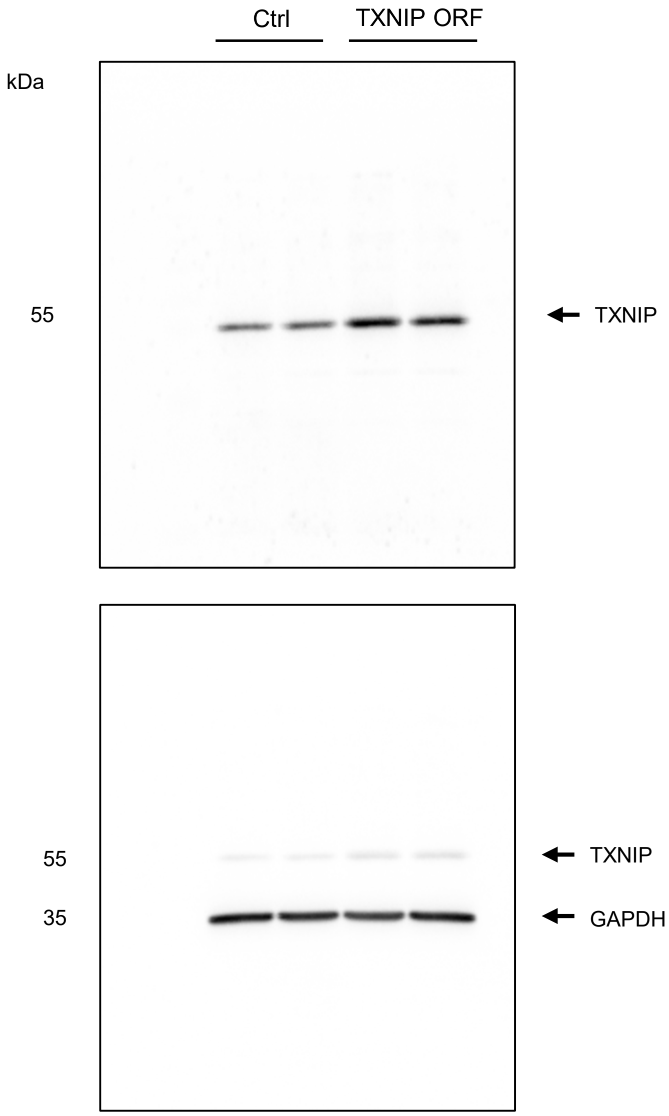

Quantification	Ctrl	Ctrl	TXNIP ORF	TXNIP ORF
TXNIP	13,464	14,068	26,464	23,665
GAPDH	17,272	14,754	13,499	17,568
Ratio	0,78	0,95	1,96	1,35

Supplementary Figure 10
